# Allosteric regulation of pyruvate kinase enables efficient and robust gluconeogenesis by preventing metabolic conflicts and carbon overflow

**DOI:** 10.1128/msystems.01131-24

**Published:** 2025-01-28

**Authors:** Fukang She, Brent W. Anderson, Daven B. Khana, Shenwei Zhang, Wieland Steinchen, Danny K. Fung, Nathalie G. Lesser, Lauren N. Lucas, David M. Stevenson, Theresa J. Astmann, Gert Bange, Jan-Peter van Pijkeren, Daniel Amador-Noguez, Jue D. Wang

**Affiliations:** 1 Department of Bacteriology, University of Wisconsin-Madison, Madison, Wisconsin, USA; 2 Department of Food Science, University of Wisconsin-Madison, Madison, Wisconsin, USA; 3 Philipps-University-Marburg, Center for Synthetic Microbiology (SYNMIKRO) & Faculty of Chemistry, Marburg, Germany; Agroscope Standort Reckenholz, Zurich, Switzerland

**Keywords:** pyruvate kinase, gluconeogenesis, overflow metabolism, allosteric regulation

## Abstract

Gluconeogenesis, the reciprocal pathway of glycolysis, is an energy-consuming process that generates glycolytic intermediates from non-carbohydrate sources. In this study, we demonstrate that robust and efficient gluconeogenesis in bacteria relies on the allosteric inactivation of pyruvate kinase, the enzyme responsible for the irreversible final step of glycolysis. Using the model bacterium *Bacillus subtilis* as an example, we discovered that pyruvate kinase activity is inhibited during gluconeogenesis via its extra C-terminal domain (ECTD), which is essential for autoinhibition and metabolic regulation. Physiologically, a *B. subtilis* mutant lacking the ECTD in pyruvate kinase displayed multiple defects under gluconeogenic conditions, including inefficient carbon utilization, slower growth, and decreased resistance to the herbicide glyphosate. These defects were not caused by the phosphoenolpyruvate–pyruvate–oxaloacetate futile cycle. Instead, we identified two major metabolic consequences of pyruvate kinase dysregulation during gluconeogenesis: failure to establish high phosphoenolpyruvate (PEP) concentrations necessary for robust gluconeogenesis and increased carbon overflow into the medium. *In silico* analysis revealed that, in wild-type cells, an expanded PEP pool enabled by pyruvate kinase inactivation is critical for maintaining the thermodynamic feasibility of gluconeogenesis. Additionally, we discovered that *B. subtilis* exhibits glyphosate resistance specifically under gluconeogenic conditions, and this resistance depends on the PEP pool expansion resulting from pyruvate kinase inactivation. Our findings underscore the importance of allosteric regulation during gluconeogenesis in coordinating metabolic flux, efficient carbon utilization, and antimicrobial resistance.

**IMPORTANCE:**

Pyruvate kinase catalyzes the final irreversible step in glycolysis and is commonly thought to play a critical role in regulating this pathway. In this study, we identified a constitutively active variant of pyruvate kinase, which did not impact glycolysis but instead led to multiple metabolic defects during gluconeogenesis. Contrary to conventional understanding, these defects were not due to the phosphoenolpyruvate–pyruvate–oxaloacetate futile cycle. Our findings suggest that the defects arose from an insufficient buildup of the phosphoenolpyruvate pool and an increase in carbon overflow metabolism. Overall, this study demonstrates the essential role of pyruvate kinase allosteric regulation during gluconeogenesis in maintaining adequate phosphoenolpyruvate levels, which helps prevent overflow metabolism and enhances the thermodynamic favorability of the pathway. This study also provides a novel link between glyphosate resistance and gluconeogenesis.

## INTRODUCTION

Regulation of central carbon metabolism in microbes allows high efficiency usage of diverse carbon sources (1). Central carbon metabolism involves glycolysis, gluconeogenesis, the pentose phosphate pathway, and the tricarboxylic acid (TCA) cycle. Glycolysis breaks glucose into two molecules of pyruvate, generating ATP and NADH. It also generates glycolytic intermediates that are essential for nucleotide and amino acid synthesis. In contrast, gluconeogenesis operates in the opposite direction to glycolysis and is required when carbon sources feeding into the early steps of glycolysis are not available. Gluconeogenesis produces essential glycolytic intermediates from non-sugar substrates, including organic acids (e.g., malate, succinate, fumarate, pyruvate) and amino acids, by consuming energy generated from other pathways such as the TCA cycle.

Gluconeogenesis shares many enzymes with glycolysis for catalyzing reversible reactions, but it bypasses the irreversible reactions using different enzymes (2). Shutting down irreversible glycolysis-specific reactions to avoid energy-wasting futile cycles is proposed to be crucial for gluconeogenesis (3, 4); however, experimental evidence remains scarce. The enzyme pyruvate kinase (PK, EC 2.1.7.40) catalyzes the irreversible last step of glycolysis by transferring the phosphate from phosphoenolpyruvate (PEP) to ADP to generate ATP and pyruvate (5). Pyruvate kinase has long been proposed to be inhibited during gluconeogenesis to avoid an energy-wasting futile cycle (6, 7), yet in bacteria it remains highly expressed during gluconeogenesis (8). *In vitro*, pyruvate kinase is allosterically regulated by metabolic effectors, such as fructose 1,6-bisphosphate (FBP) (9, 10), AMP (10–15), ribose 5-phosphate (R5P) (10, 11, 14, 15), glucose 6-phosphate (G6P) (10, 12, 13), and glycerol 3-phosphate (G3P) (10). Allosteric regulations of pyruvate kinase are believed to play a critical role during glycolysis in mammalian and yeast systems, e.g., in tumorigenesis (16). In contrast, the physiological effects of pyruvate kinase regulation in bacteria are poorly understood due to a lack of dysregulated mutants.

In this study, we identified an autoinhibitory role of an extra C-terminal domain in allosteric regulation of pyruvate kinase from the bacterial phylum Firmicutes (recently renamed Bacillota) (14, 17). Dysregulating pyruvate kinase with a domain-truncation mutant results in a constitutively active enzyme, enabling us to assess its physiological effects. Notably, this mutant did not exhibit any distinct phenotypes during growth on glucose; however, it manifested broad fitness defects specifically during growth on gluconeogenic substrates. These defects are not linked to a futile cycle at the PEP–pyruvate–OAA junction. Instead, they stem from an inability to accumulate sufficient levels of PEP, which, according to our analysis, is required to alleviate thermodynamic bottlenecks during gluconeogenesis. Furthermore, under gluconeogenic conditions, elevated levels of PEP confer resistance to the herbicide glyphosate, which competes with PEP. This resistance is lost in the mutant. We propose that high PEP levels, enabled by allosteric regulation of pyruvate kinase, are critical for thermodynamically favorable gluconeogenesis and antimicrobial resistance.

## RESULTS

### The extra C-terminal domain (ECTD) is required for the inhibition of *B. subtilis* pyruvate kinase activity by metabolic effectors *in vitro*

**Fig 1:**
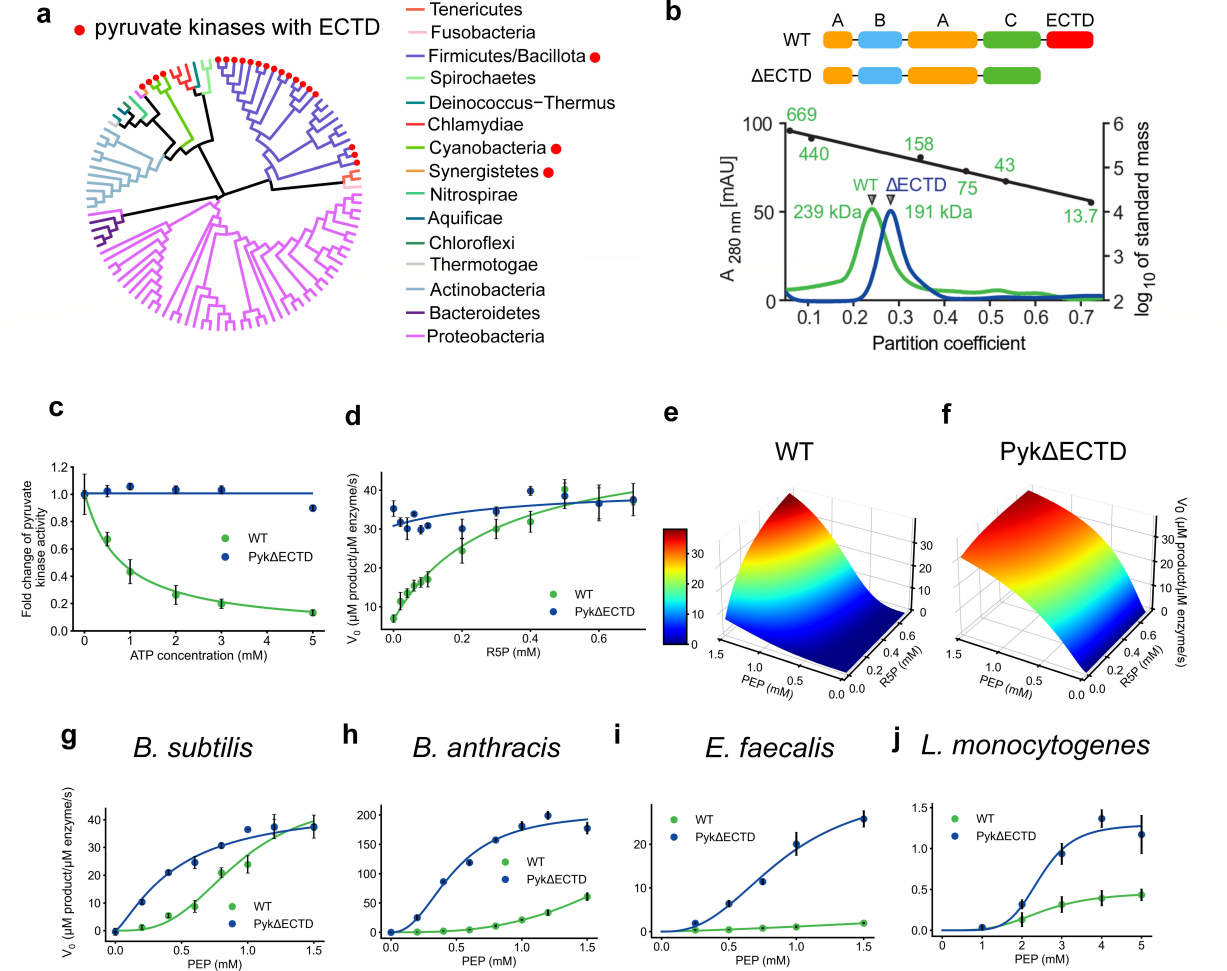
*In vitro* characterizations of enzymatic activities of full-length pyruvate kinase (WT) and the ΔECTD variant (PykΔECTD). (**a**) Pyruvate kinases across a phylogenetic tree of bacterial reference genomes. Red dots indicate the presence of ECTD. Detailed species list is presented in Table S1. (**b**) Size-exclusion chromatography of wild-type (WT, green trace) and ΔECTD pyruvate kinase (blue trace) with apparent molecular masses of approximately 239 and 191 kDa, respectively. These correspond to the theoretical masses of 258.0 (monomeric mass of 64.5 kDa) and 212.8 kDa (monomeric mass of 53.2 kDa) for homoterameric wild-type and ΔECTD pyruvate kinase. The black trace represents the average partition coefficients and linear regression thereof of a protein standard containing thyroglobulin (669 kDa), ferritin (440 kDa), aldolase (158 kDa), conalbumin (75 kDa), ovalbumin (43 kDa), and RNase A (13.7 kDa). (**c**) *B. subtilis* pyruvate kinase wild type (WT) and ΔECTD in the presence of indicated concentrations of ATP. *y*-axis: V_0_ normalized against the absence of ATP. Error bars represent the standard error of the mean (*n* = 3). (**d**) *B. subtilis* pyruvate kinase activity of wild-type and ΔECTD pyruvate kinase with indicated concentrations of R5P. Data were fitted to a nonessential activation equation (solid curves). (**e and f**) Three dimensional fit of *B. subtilis* wild-type (**e**) and ΔECTD (**f**) pyruvate kinase activities in the presence of combinations of different concentrations of the substrate PEP and activator R5P. The original plot is in Fig. S3c and d. (**g–j**) Pyruvate kinase activities from Bacillota species with indicated concentrations of PEP (*x*-axis). Data were fitted to a nonessential activation equation or an allosteric sigmoidal equation (**h–j**) (solid curves).

Pyruvate kinase is a key glycolytic enzyme that shares conserved architecture of three principal domains (A, B, and C domains) from bacteria to eukaryotes. In many bacterial species, pyruvate kinase also harbors an extra C-terminal domain (ECTD) (Fig. S1), which has been given various designations in previous studies, including ECTD, CT, ECTS, C′ domain, and PEPut (7, 17–19). We performed phylogenetic analysis of pyruvate kinase sequences from 113 NCBI reference genomes, as we have done previously (20), revealing that pyruvate kinases with an ECTD are conserved in the Bacillota phylum (except for *Streptococcus* species) and parts of Cyanobacteria phylum (Fig. 1a; Table S1).

We investigated the impact of the pyruvate kinase ECTD using *B. subtilis*, a well-established model bacterium within the *Bacillota* phylum. We recombinantly expressed and purified both the wild-type full length pyruvate kinase (WT Pyk) and a truncated variant lacking the ECTD domain (PykΔECTD) (Fig. S2). Both the wild-type and truncated pyruvate kinase formed classical homotetramers in solution (7), indicating that the ECTD is dispensable for pyruvate kinase oligomerization (Fig. 1b).

Next, we measured pyruvate kinase activity using a classical coupled enzyme assay (21). We first measured pyruvate kinase activity across various concentrations of its inhibitor, ATP (15). Wild-type *B. subtilis* pyruvate kinase activity was significantly inhibited by ATP (<15% remaining at 5 mM ATP) (Fig. 1c). Given that ATP concentrations in *B. subtilis* cells typically reach around 3–5 mM under most growth conditions (e.g., Table S2), all subsequent pyruvate kinase assays were conducted in the presence of 5 mM ATP.

Additionally, wild-type *B. subtilis* pyruvate kinase was activated by AMP or R5P (Fig. S3a), which are known activators of previously characterized pyruvate kinases (14, 15). In contrast, the pyruvate kinase variant lacking ECTD maintained enzymatic activity but showed no response to either the inhibitor ATP (Fig. 1c), or the activators AMP and R5P (Fig. 1d; Fig. S3b). This suggests that the ECTD domain is essential for regulating pyruvate kinase activity.

To systematically and quantitatively understand pyruvate kinase regulation by metabolic effectors, we titrated both the substrate PEP and the activator R5P (14) while keeping the inhibitor ATP at 5 mM. Pyruvate kinase activity was then measured for each combination of substrate and activator concentrations. Three-dimensional plots were generated with substrate concentration on the *x*-axis, activator concentration on the *y*-axis, and reaction rate on the *z*-axis (Fig. 1e and f). The results revealed that wild-type pyruvate kinase exhibits a classical sigmoidal response to the substrate PEP (7), with a Hill coefficient of 3.1, and is allosterically activated by R5P with a *K*_*A*_ of 0.29 mM (Fig. 1e; Fig. S3c; Table 1). In contrast, the pyruvate kinase variant lacking the ECTD domain shows activity even without the activator R5P and displays a Hill coefficient of 1.3 with respect to PEP (Fig. 1f; Fig. S3d; Table 1).

**TABLE 1:** Enzyme kinetics parameters of *B. subtilis* pyruvate kinase

Parameter	WT	PykΔECTD
*k*_cat_	54.1 ± 3.8	53.8 ± 3.1
*K*′_PEP^*a*^	0.84 ± 0.25 mM	0.32 ± 0.11 mM
Hill coefficient PEP	3.1	1.3
*K*_*m*__ADP^*b*^	1.01 ± 0.03 mM	1.83 ± 0.24 mM
*K*_*A*__R5P^*c*^	0.57 ± 0.04 mM	N.A.^*e*^
*K*_*i*__ATP^*d*^	0.52 ± 0.05 mM	N.A.^*e*^

^*a*^ Apparent Michaelis constant for the substrate PEP in the allosteric sigmoidal model of pyruvate kinase.

^*b*^ Michaelis constant of pyruvate kinase when using ADP as the substrate.

^*c*^ Equilibrium constant of R5P disassociation, defined as the ratio of [*E*][*A*]/[EA] at equilibrium, where [*E*] is the enzyme concentration, [*A*] is the activator concentration, and [EA] is the activator-bound enzyme concentration.

^*d*^ Inhibition constant of ATP, defined as the ratio of [*E*][*I*]/[EI] at equilibrium, where [*E*] is the enzyme concentration, [*I*] is the inhibitor concentration, and [EI] is the inhibitor-bound enzyme concentration.

^*e*^ PykΔECTD is not regulated by R5P or ATP. Data cannot be fitted to get a reasonable parameter.

To investigate whether the autoinhibitory role of the pyruvate kinase ECTD observed in *B. subtilis* is conserved across other Bacillota species, we purified pyruvate kinases and truncated ΔECTD variants from several representative species, including *Bacillus anthracis*, *Listeria monocytogenes*, and *Enterococcus faecalis*, and measured their activities. Although we observed that pyruvate kinases from different species showed varied responses to inhibitors and activators, the commonality (shown in Fig. 1g through j) is that wild-type pyruvate kinases were consistently more inhibited compared with truncated ΔECTD variants within each species. These findings suggest that the ECTD domain acts as an autoinhibitory domain in representative Bacillota species, including *B. subtilis*, *B. anthracis, L. monocytogenes*, and *E. faecalis*.

### The extra C-terminal domain (ECTD) of pyruvate kinase is required for the inhibition of pyruvate kinase activity *in vivo*

**Fig 2:**
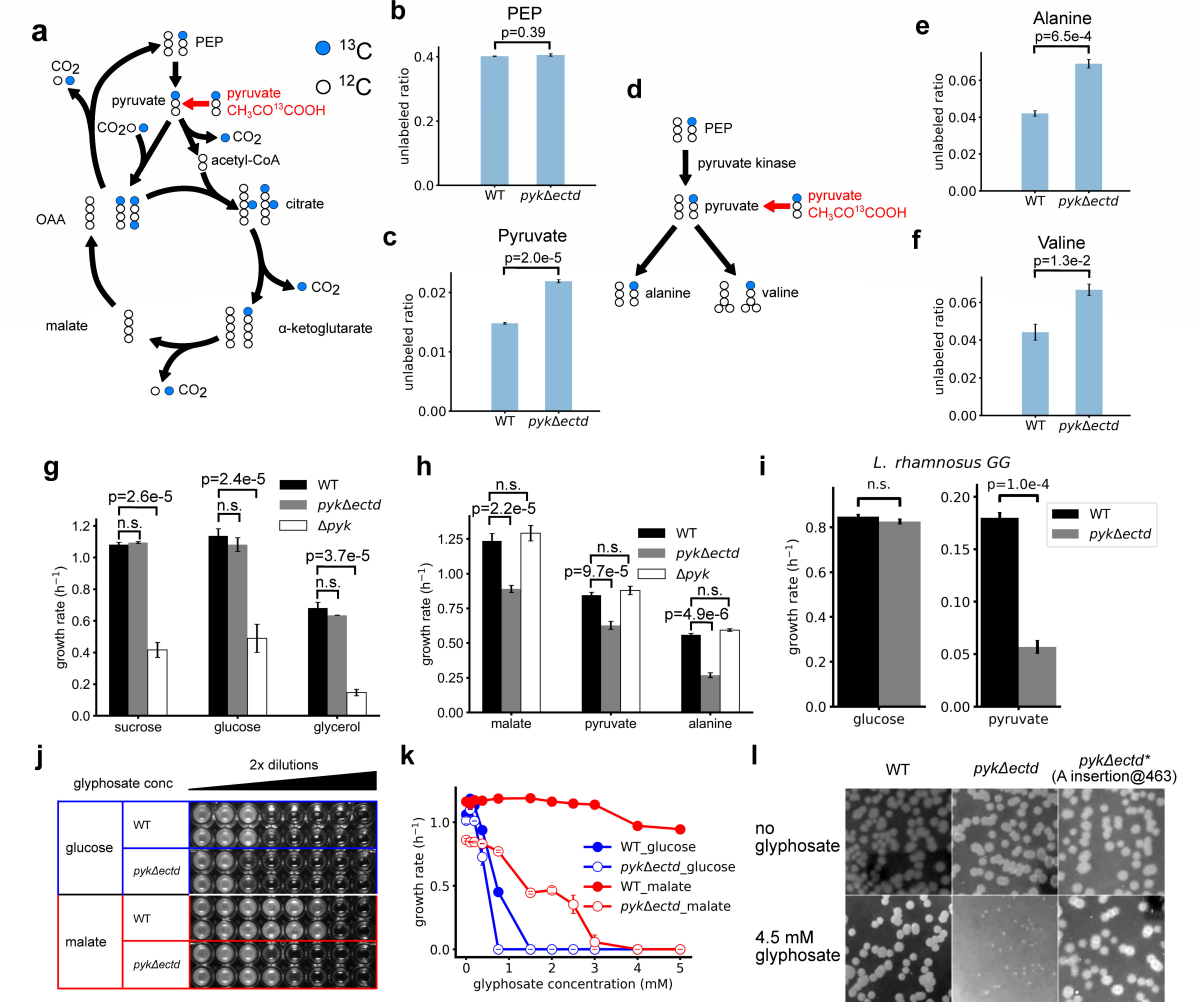
Metabolic flux, growth rate, and glyphosate resistance in full length (WT) and pyruvate kinase mutant lacking ECTD (*pyk*Δ*ectd*) during glycolysis and gluconeogenesis. (**a**) Schematics for quantification of pyruvate kinase activity *in vivo* by labeling metabolites in cells grown with 1-^13^C-labeled pyruvate as the sole carbon source. OAA: oxaloacetate. (**b**) Unlabeled ratio of PEP (over total PEP) in wild-type (WT) and *pyk*Δ*ectd* cells. (**c**) Unlabeled ratio of pyruvate (over total intracellular pyruvate) in *pyk*Δ*ectd* and wild-type cells. (**d**) Schematics of ^13^C labeling of alanine and valine as a proxy of the intracellular pyruvate ^13^C labeling. (**e and f**) Unlabeled ratios of (**f**) alanine and (**f**) valine in *pyk*Δ*ectd* and WT cells in [1-^13^C] pyruvate media. (**g and h**) Steady state growth rates of wild-type (WT), *pyk*Δ*ectd*, and Δ*pyk* mutants in indicated glycolytic media (**g**) and gluconeogenic media (**h**). *P* values are obtained by F test followed by Tukey’s HSD test; n.s.: *P* > 0.05. (**i**) Steady state growth rates of *L. rhamnosus* GG *pyk*Δ*ectd* mutant and wild type in glucose and pyruvate media. (**j**) Minimal inhibitory concentration (MIC) of glyphosate for WT and *pyk*Δ*ectd* cells in glucose or malate media. Glyphosate concentrations were 0.1875, 0.375, 0.75, 1.5, 3, 6, 12, and 24 mM from left to right. (**k**) Growth rates of WT and *pyk*Δ*ectd* mutants in media with glucose or malate as the sole carbon source under different concentrations of glyphosate. Error bars represent standard error of the mean. (**L**) Colonies of WT, *pyk*Δ*ectd*, and a spontaneous suppressor on agar plates supplemented with glyphosate.

Our enzymatic data show that pyruvate kinase ECTD is required for the regulation of pyruvate kinase activity by metabolic effectors *in vitro*. To evaluate the effect of ECTD in bacterial cells, we engineered a *B. subtilis* mutant by replacing its wild-type pyruvate kinase gene (*pyk*) at its endogenous locus with a truncated *pyk* mutant retaining residues 1–474 but lacking ECTD (*pyk*Δ*ectd*). We then performed isotope tracing experiments using [1-^13^C] pyruvate as the sole carbon source (Fig. 2a). Cellular pyruvate can be derived either from external ^13^C-labeled pyruvate or from PEP via pyruvate kinase. Since PEP can be produced from both labeled oxaloacetate (OAA) from pyruvate and unlabeled OAA from the TCA cycle, increased pyruvate kinase activity would result in a higher concentration of unlabeled pyruvate in the cell.

By analyzing the isotope composition of primary metabolites in cells grown on [1-^13^C] pyruvate (Fig. 2a), we compared the *in vivo* activities of wild-type and ΔECTD pyruvate kinase (Fig. 2b through f; Fig. S4b). We found that the proportion of unlabeled PEP was similar in both wild-type and mutant cells (Fig. 2b). However, the *pyk*Δ*ectd* mutant had a higher proportion of unlabeled pyruvate compared with wild-type cells (Fig. 2c), indicating higher pyruvate kinase activity in the *pyk*Δ*ectd* mutant. To rule out complications from pyruvate in the media being carried over during extraction, we also examined the isotope composition of alanine and valine, whose carbon backbones are derived entirely from pyruvate (Fig. 2d). Both alanine and valine in *pyk*Δ*ectd* cells had less ^13^C labeling than wild-type cells (Fig. 2e and f), confirming that pyruvate kinase activity is higher in the *pyk*Δ*ectd* mutant.

### ECTD domain of pyruvate kinase promotes growth specifically during gluconeogenesis

Pyruvate kinase regulation by metabolic effectors has been proposed as a key mechanism for maintaining fitness during glycolysis and gluconeogenesis, but experimental evidence is lacking due to inability to engineer dysregulated pyruvate kinase mutants. The *pyk*Δ*ectd* mutant provides a crucial tool to evaluate the physiological consequences of failed pyruvate kinase inhibition. We first screened phenotypes of the *pyk*Δ*ectd* mutant on different carbon sources using Biolog Phenotype MicroArrays, which measure cell respiration as a reporter for growth (Fig. S5a). Intriguingly, the *pyk*Δ*ectd* mutant exhibited reduced total cell respiration rates on gluconeogenic carbon sources (such as pyruvate and malate) but not on glycolytic carbon sources (such as glucose and fructose) (Fig. S5b). The reduction in respiration can be explained by slower growth.

To test this hypothesis, we directly measured the growth curves of *B. subtilis* cells and compared the exponential growth rates of wild-type pyruvate kinase (WT), pyruvate kinase deletion (Δ*pyk*), and the *pyk*Δ*ectd* mutant on either glycolytic (sucrose, glucose, and glycerol) or gluconeogenic (pyruvate, malate, and alanine) carbon sources (Fig. 2g and h). The Δ*pyk* mutant grew significantly slower than wild-type cells during glycolytic growth (Fig. 2g), which is expected as pyruvate kinase activity is crucial for glycolysis. During growth on gluconeogenic carbon sources, the Δ*pyk* mutant grew similarly to wild-type cells, confirming that pyruvate kinase activity is dispensable during gluconeogenesis (Fig. 2h).

In contrast to Δ*pyk,* there was no difference in growth rates between the wild-type and *pyk*Δ*ectd* strains during glycolysis, suggesting that pyruvate kinase regulation activity during glycolysis does not provide a fitness benefit. Strikingly, the *pyk*Δ*ectd* mutant grew significantly slower than wild type during gluconeogenesis (Fig. 2h), indicating that pyruvate kinase regulation is crucial for gluconeogenesis.

Next, we performed complementation studies by overexpressing wild-type *pyk* from an ectopic locus, which successfully rescued the growth defect of the Δ*pyk* mutant during glycolysis (Fig. S6a) but did not rescue the growth defect of the *pyk*Δ*ectd* mutant during gluconeogenesis (Fig. 6b and c), confirming that *pyk*Δ*ectd* is a dominant allele. Overall, the results suggest that the constitutively active pyruvate kinase leads to slow gluconeogenic growth.

Pyruvate kinase ECTD shares homology with the swiveling domains (Pfam PF00391) (18) of the pyruvate phosphate dikinase and several PEP-utilizing enzymes (22) that contains a conserved histidine residue reported to be responsible for phosphorelay (23–25). This histidine (H539 in *B. subtilis*) is conserved in the Bacillota phylum with the exception of *S. aureus* (Fig. S1). Another potential phosphorylation site is threonine 537 on *B. subtilis* pyruvate kinase ECTD (19, 26). We found that substituting either histidine 539 or threonine 537 to alanine, which prevents potential phosphorylation of these residues, did not result in growth defect as in the *pyk*Δ*ectd* mutant (Fig. S6d). This result suggests that regulation of pyruvate kinase by its ECTD is unlikely through phosphorylation of these conserved residues.

To test whether pyruvate kinase ECTD plays a similar role in Bacillota beyond *B. subtilis*, we deleted the ECTD domain of pyruvate kinase in the probiotic Bacillota *Lacticaseibacillus rhamnosus* GG (27). The resulting *L. rhamnosus* GG *pyk*Δ*ectd* mutant grew similarly to wild-type cells in glucose medium but displayed a growth defect in pyruvate medium (Fig. 2i; Fig. S7), confirming that the pyruvate kinase ECTD domain also promotes gluconeogenic growth in *L. rhamnosus*.

### Bacteria display higher resistance to the herbicide glyphosate during gluconeogenesis than glycolysis dependent on pyruvate kinase ECTD

In addition to its impact on growth rate, we observed that the pyruvate kinase ECTD also enhances resistance to glyphosate, the active ingredient in the herbicide Roundup (28) and a potent antimicrobial (29). Glyphosate competes with the glycolytic intermediate PEP to inhibit 5-enolpyruvylshikimate 3-phosphate (EPSP) synthase and downstream aromatic amino acid synthesis (30, 31) (Fig. S8a). *B. subtilis*, a soil bacterium, was sensitive to glyphosate when grown in glycolytic media, as reported previously (29). However, to our surprise, *B. subtilis* displayed much higher resistance to glyphosate when the gluconeogenic malate was supplemented as the sole carbon source (Fig. 2j and k). Importantly, the protective effect of gluconeogenesis was strongly dependent on the ECTD domain of the pyruvate kinase. The minimal inhibitory concentration (MIC) of glyphosate for *pyk*Δ*ectd* cells was fourfold lower than that for wild-type cells (Fig. 2j), and the *pyk*Δ*ectd* mutant displayed strongly reduced growth rates compared with wild-type cells (Fig. 2k). While wild-type cells formed many colonies on solid media supplemented with 4.5 mM glyphosate and malate, *pyk*Δ*ectd* mutant cells formed no colonies, except for a single colony. Upon re-inoculation, this single colony showed increased resistance to glyphosate, suggesting it was a suppressor (Fig. 2l; Fig. S8b). Whole genome sequencing of this suppressor revealed a single frameshift mutation in the *pyk* locus at nucleotide position 463 (amino acid 155), resulting in a premature stop codon at amino acid 159 upstream of the active site (Fig. S1). This suggests that the loss of function of pyruvate kinase suppresses the glyphosate sensitivity of *pyk*Δ*ectd* mutants. Thus, *B. subtilis* withstands glyphosate toxicity by enabling the negative regulation of pyruvate kinase activity through its ECTD.

### The pyruvate kinase ECTD domain enhances carbon usage efficiency by preventing carbon overflow during gluconeogenesis

**Fig 3:**
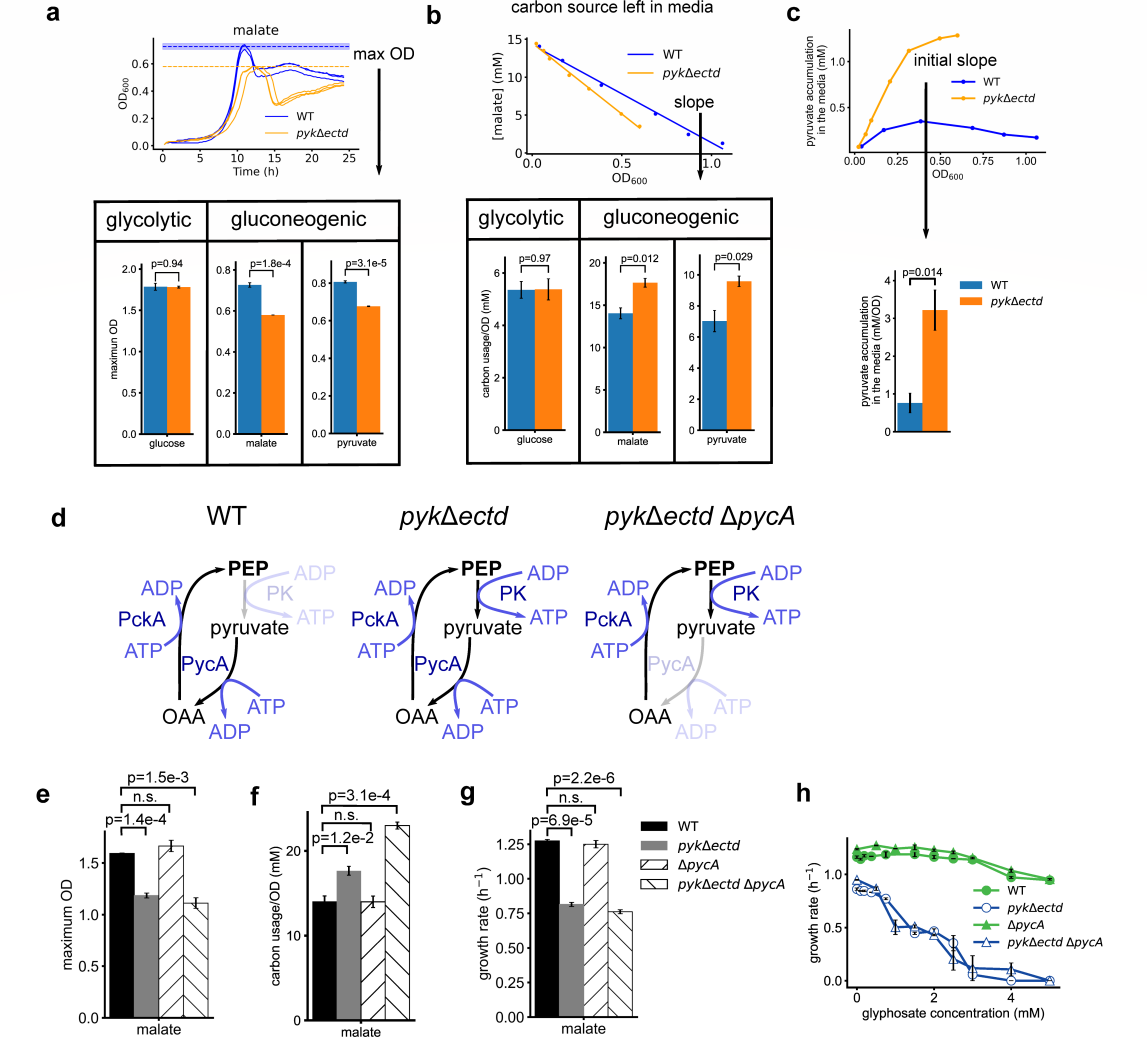
Loss of pyruvate kinase ECTD leads to low carbon use efficiency independent of the futile cycle during gluconeogenesis. (**a**) Top: examples of growth curves of *pyk*Δ*ectd* and WT cells on malate as the sole carbon source. Botton: Maximum OD in media with 0.2% (wt/vol) glucose, malic acid, or sodium pyruvate as the sole carbon sources. (**b**) *pyk*Δ*ectd* cells consumed more gluconeogenic carbon sources than wild-type cells per OD increase. Carbon source consumption rate was measured with HPLC or LC-MS. (**c**) Overflow of pyruvate in the media when *pyk*Δ*ectd* mutant or WT cells grew with malate as the sole carbon source. Pyruvate concentration in the media was measured with LC-MS and calibrated based on standard curve. (**d**) Schematics of how the PEP–pyruvate–oxaloacetate node form a potential futile cycle in *pyk*Δ*ectd* mutant during gluconeogenic growth. Simultaneous activation of pyruvate kinase (PK), pyruvate carboxylase (PycA), and PEP carboxykinase (PckA) would consume one molecule of ATP every cycle without product accumulation. (**e–h**) *pycA* deletion, which would disrupt the potential futile cycle, does not rescue the *pyk*Δ*ectd* defect in maximum OD (**e**), carbon usage efficiency (**f**), growth rates, and glyphosate resistance (**h**).

In addition to reduced growth rates, we also noticed a reduction in maximum optical density in the *pyk*Δ*ectd* mutant during gluconeogenic growth. When compared with wild type, the *pyk*Δ*ectd* mutant had 25% and 19% reduced maximal optical density at 600 nm (OD_600_) when grown in the gluconeogenic substrates malate or pyruvate (Fig. 3a; Fig. S9c and d) but not in the glycolytic carbon source glucose (Fig. 3a; Fig. S9a and b). This suggests that *pyk*Δ*ectd* cells are less efficient in synthesizing biomass from gluconeogenic carbon sources compared with wild-type cells.

To directly measure carbon utilization efficiency, we quantified the consumption of carbon sources per unit of cell growth. We cultured wild-type and *pyk*Δ*ectd* cells in media with glucose, malate, or pyruvate as the sole carbon source. During steady state growth, we used HPLC and LC-MS to measure the remaining carbon sources in the media (Fig. S10). The results showed that *pyk*Δ*ectd* cells utilized glucose as efficiently as wild-type cells but consumes more malate and pyruvate per unit of cell growth (Fig. 3b). These findings confirm that *pyk*Δ*ectd* cells have lower carbon use efficiency, specifically during gluconeogenesis.

The reduced carbon use efficiency could be due to a futile carbon usage cycle, carbon overflow, or both. To test for carbon overflow, we measured pyruvate excretion into the culture media using LC-MS (Fig. 3c). We found that *pyk*Δ*ectd* mutant excreted significantly more pyruvate and at a much higher rate (~4×) compared with wild-type cells. This overflow phenomenon likely contributes to the reduced efficiency of *pyk*Δ*ectd* cells in utilizing gluconeogenic carbon sources.

### The futile cycle does not contribute to the fitness defects of *pyk*Δ*ectd* during gluconeogenesis

A long-standing hypothesis is that pyruvate kinase is inhibited during gluconeogenesis to avoid an energy-wasting futile cycle (7). In *B. subtilis*, pyruvate is converted to OAA by PycA (pyruvate carboxylase), then OAA is converted to PEP by PckA (PEP carboxykinase) (32), with both enzymes being active during gluconeogenesis. In this case, failure to inhibit pyruvate kinase during gluconeogenesis would lead to PEP being converted back to pyruvate, forming a futile cycle that consumes ATP without product accumulation (Fig. 3d). To test whether this futile cycle hypothesis contributes to the gluconeogenic defects of *pyk*Δ*ectd*, we deleted *pycA* in the *pyk*Δ*ectd* mutant to disrupt the futile cycle (Fig. 3d). We assessed carbon utilization efficiency, growth rate, and glyphosate resistance in wild-type, *pyk*Δ*ectd*, Δ*pycA*, and Δ*pycA pyk*Δ*ectd* double mutant in media with malate as the sole carbon source (Fig. 3e through h). Interestingly, Δ*pycA* did not rescue any of the defects introduced by *pyk*Δ*ectd* mutant. This indicates that the futile cycle does not contribute to the fitness defect of pyruvate kinase dysregulation during gluconeogenesis.

### Metabolomic analysis highlights the role of pyruvate kinase regulation in PEP pool expansion during gluconeogenesis

**Fig 4:**
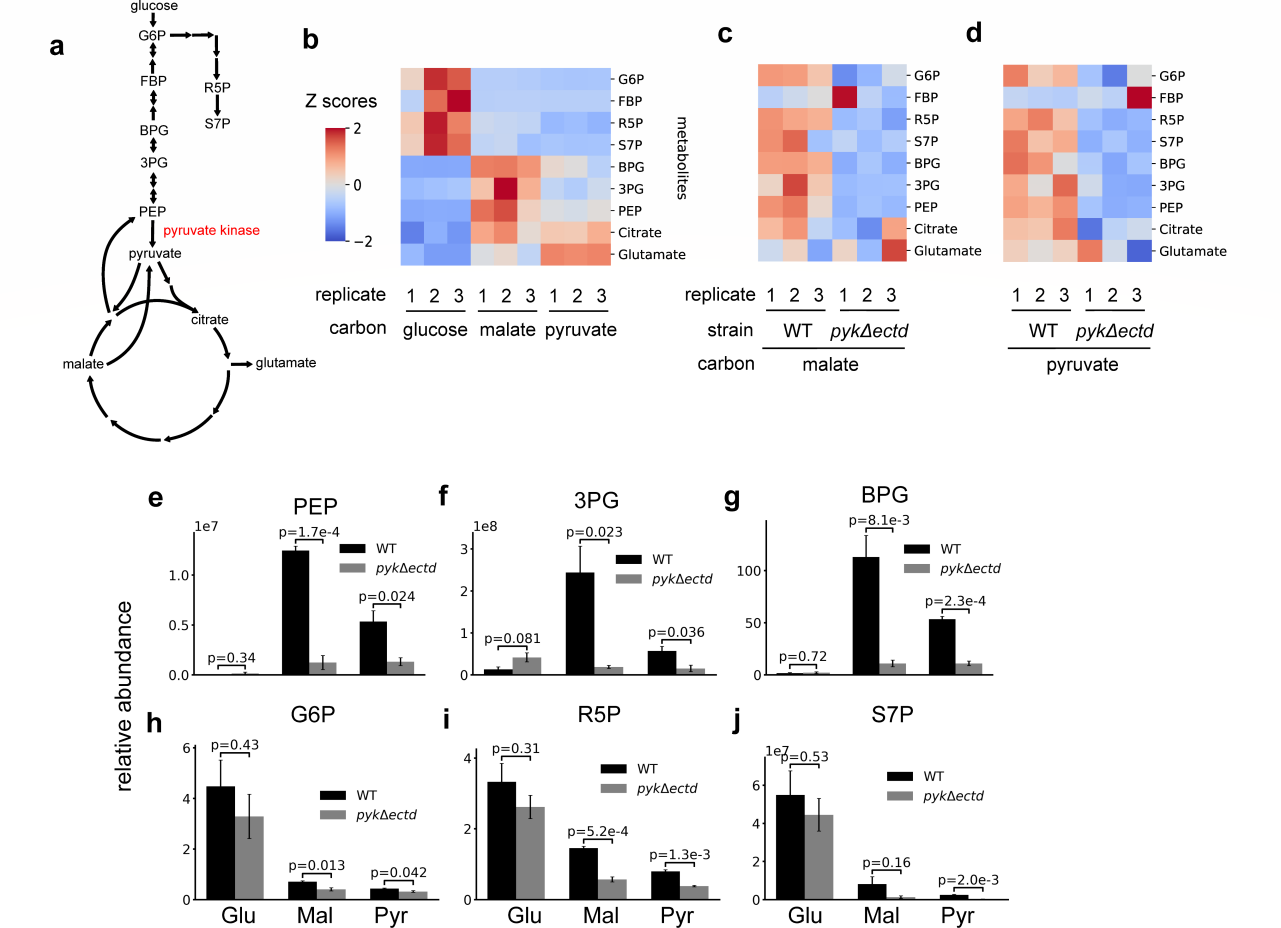
Pyruvate kinase dysregulation leads to failure to expand PEP pool. (**a**) Schematics of gluconeogenesis, part of the pentose phosphate pathway, and the TCA cycle. Important target metabolites are indicated. G6P: glucose 6-phosphate; FBP: fructose 1,6-bisphosphate; R5P: ribose 5-phosphate; S7P: sedoheptulose 7-phosphate; BPG: 1,3-bisphosphoglycerate; 3 PG: 3-phosphoglycerate. (**b**) Heatmaps of intracellular metabolites in wild-type cells grown in glucose, malate or pyruvate carbon sources, as indicated. Z-score is obtained for each metabolites by subtracting the mean data from all three carbon sources and dividing by the standard deviation. (**c and d**) Heatmaps of intracellular metabolites in wild-type and *pyk*Δ*ectd* cells grown in gluconeogenic sources (**c**) malate and (**d**) pyruvate. Z-score is obtained for each metabolite by subtracting the mean data from both WT and mutant and dividing by the standard deviation. (**e**-**j**) Relative intracellular abundance of metabolites upstream and downstream of pyruvate kinase. See Materials and Methods: Metabolic analysis by LC-MS.

To understand the overall impact of pyruvate kinase regulation on central metabolism (Fig. 4a), we cultured wild-type and *pyk*Δ*ectd* mutant under both glycolytic and gluconeogenic conditions and monitored central carbon metabolites using LC-MS (Fig. 4; Fig. S11; Table S3). In wild type, we found that cells grown in glucose exhibited significantly higher intracellular levels of upper glycolytic intermediates (e.g., G6P, FBP) and pentose phosphate pathway intermediates (e.g., R5P, S7P) compared with cells grown on malate or pyruvate (Fig. 4b). Conversely, the levels of lower glycolytic intermediates (e.g., BPG, 3 PG, PEP), TCA cycle intermediates (e.g., citrate), and amino acids derived from the TCA cycle (e.g., glutamate) were notably higher in cells grown on malate or pyruvate compared with glucose (Fig. 4b).

For the *pyk*Δ*ectd* mutant, cells grown in glucose displayed comparable central metabolism as the wild type (Fig. S11). However, major differences were observed between the mutant and wild-type cells during growth on glucogenic carbon sources (Fig. 4c and d). Notably, PEP levels in the *pyk*Δ*ectd* mutant were more than three times lower than those in wild-type cells (Fig. 4e). Other lower-glycolytic intermediates (e.g. BPG, 3PG) were similarly depleted in the *pyk*Δ*ectd* mutant during gluconeogenesis, likely due to their equilibrium with PEP through reversible reactions (Fig. 4f and g). Additionally, G6P levels were reduced in *pyk*Δ*ectd* mutant compared with wild type (Fig. 4h). This decrease in G6P levels could be attributed to the role of PEP as an activator of the gluconeogenesis-specific enzyme fructose 1,6-bisphosphatase (33).

Moreover, pentose phosphate pathway intermediates (R5P, S7P) were also lower in *pyk*Δ*ectd* than in wild type (Fig. 4i and j), likely due to the diminished levels of glycolytic intermediates that feed into this pathway. These findings suggest that the inability to deactivate pyruvate kinase in the *pyk*Δ*ectd* strain during gluconeogenesis hampers PEP accumulation, negatively impacting gluconeogenic carbon flux and the generation of essential glycolytic intermediates for macromolecular biosynthesis.

### Expanded PEP pool is critical for the thermodynamic favorability of gluconeogenesis

To quantitatively determine if the reduced levels of PEP in the *pyk*Δ*ectd* mutant led to metabolic pathway bottlenecks, we integrated our metabolite concentration data with computational estimates of standard Gibbs free energies and performed a thermodynamic analysis of gluconeogenesis (34). To facilitate the modeling, we obtained the absolute intracellular metabolite concentrations using a previously described method (35) in wild-type and *pyk*Δ*ectd* mutant during growth on malate and pyruvate (Table S2). We then used the max–min driving force (MDF) computational tool (36), which identifies the most thermodynamically constraining reaction(s) in a pathway and maximizes their reduction in Gibbs free energy using calculated optimized metabolite concentrations.

**Fig 5:**
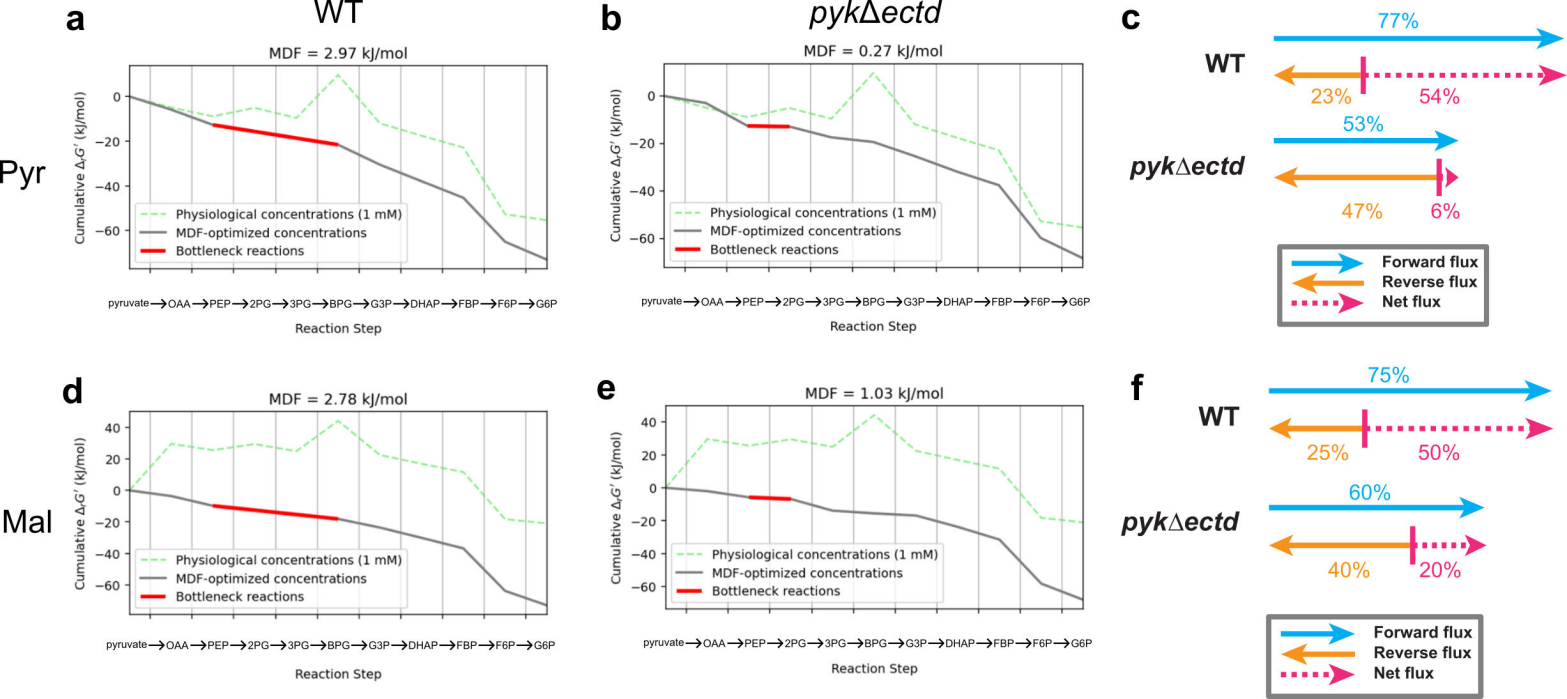
*In silico* analyses of thermodynamic feasibility of the gluconeogenesis. (**a and b**) Thermodynamic profiles for gluconeogenesis in wild-type (**a**) and *pyk*Δ*ectd* (**b**) cells grown in pyruvate. The calculated MDF (max–min driving force) value is displayed at the top of each graph. The cumulative Gibbs free energies along the gluconeogenesis are plotted. The green line represents Gibbs free energy changes calculated from the same concentration (1 mM) of all metabolites, while the black line indicates Gibbs free energy changes calculated from the MDF-optimized concentrations. Optimized MDF values were determined by searching within concentration bounds for PEP, ATP, and ADP, set at 40%, 100%, and 40% of their calculated absolute intracellular concentrations, respectively. Thermodynamic bottlenecks are highlighted in red. (**c**) The calculated forward flux ratio, reverse flux ratio, and net flux ratio of the bottleneck reaction in gluconeogenesis for wild-type and *pyk*Δ*ectd* cells grown in pyruvate. (**d and e**) *In silico* analyses of the cumulative changes in Gibbs free energy during gluconeogenesis in (**d**) wild-type and (**e**) *pyk*Δ*ectd* cells grown in malate. (**f**) The calculated forward flux ratio, reverse flux ratio, and net flux ratio of the bottleneck reaction in gluconeogenesis for wild-type and *pyk*Δ*ectd* cells grown in malate.

Our thermodynamic analyses revealed that in wild-type cells grown on pyruvate, the three reactions following PEP were identified as moderate thermodynamic bottlenecks, each with optimized free energies (MDF values) of −2.97 kJ/mol (Fig. 5a). However, in *pyk*Δ*ectd* cells grown on pyruvate, due to severely reduced levels of PEP, the reaction catalyzed by enolase (PEP to 2-phosphoglycerate) approached thermodynamic equilibrium (i.e., −0.27 kJ/mol) and became the single most constraining reaction step in gluconeogenesis (Fig. 5b).

To quantitate how this reduction in thermodynamic favorability impacts pathway flux, we used the flux-force efficacy relation (see Materials and Methods) (36):


$$\frac{J^{+}-J^{-}}{J^{+}+J^{-}}=\frac{e^{-\Delta G/RT}-1}{e^{-\Delta G/RT}+1}$$

Using this formula, we determined that the decrease in free energy drop of the enolase reaction in *pyk*Δ*ectd* cells results in a ninefold reduction (6% vs 54%) in net flux compared with the net fluxes of the bottleneck reactions in wild-type cells (Fig. 5c). Consistent with these findings, enolase also became the single most thermodynamically constraining pathway step in gluconeogenesis in mutant cells grown on malate and exhibited a similar large reduction in net flux compared with wild-type cells (Fig. 5d through f).

Taken together, these results indicate that inhibition of pyruvate kinase activity during gluconeogenesis enables intracellular PEP accumulation, which is required for thermodynamically favorable gluconeogenesis. In contrast, pyruvate kinase lacking ECTD remains active, which leads to substantially lower intracellular levels of PEP, resulting in a more thermodynamically constrained gluconeogenesis that contributes to the observed growth defects.

## DISCUSSION

**Fig 6:**
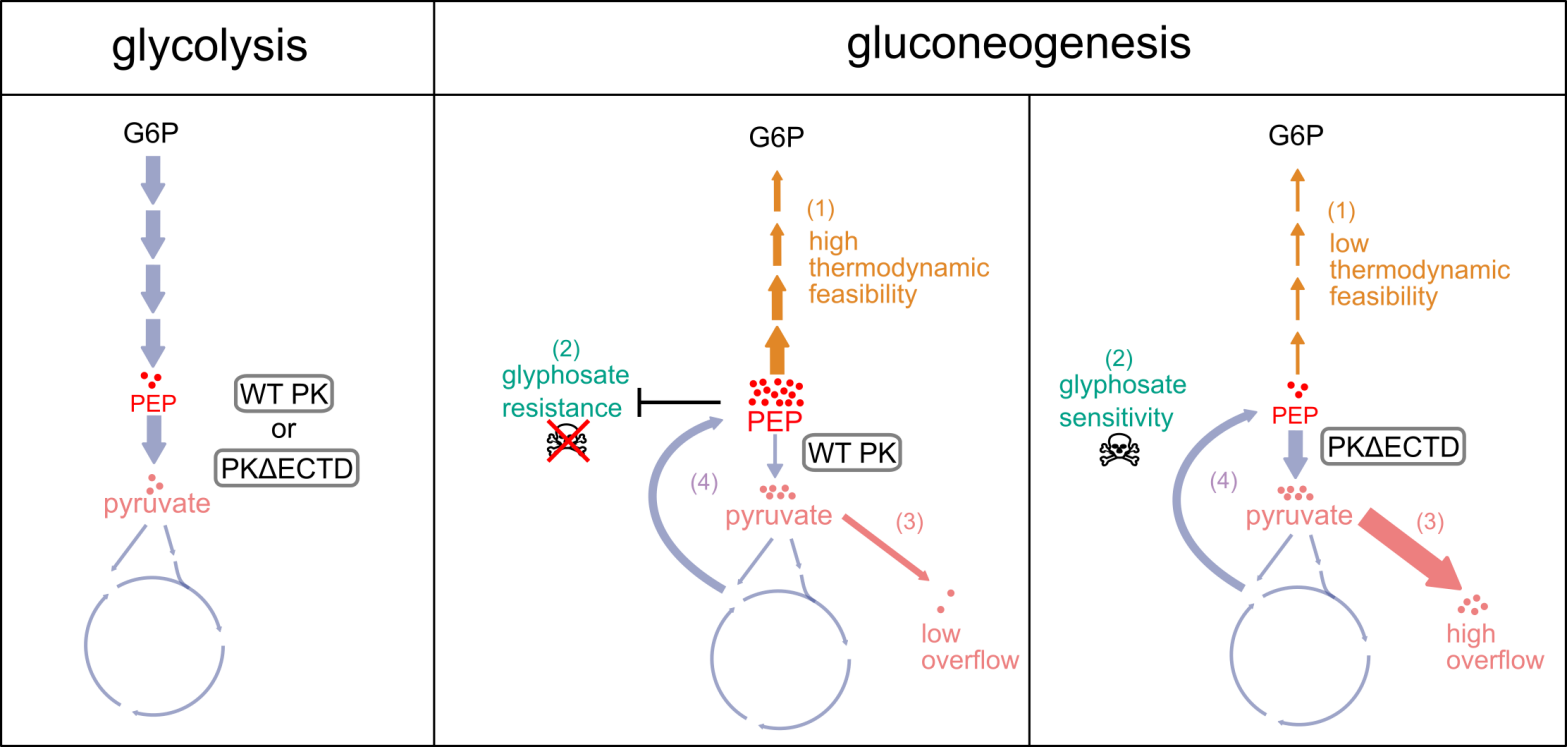
Schematic illustrating how the regulated inactivation of pyruvate kinase enables efficient gluconeogenesis. During gluconeogenesis, the inactivation of pyruvate kinase expands the PEP pool, facilitating thermodynamically favorable gluconeogenic flux (1) and enhancing resistance to glyphosate, a PEP competitor (2). Loss of this regulation by removal of pyruvate kinase ECTD domain results in reduced gluconeogenic efficiency, increased sensitivity to glyphosate, and higher carbon overflow into the extracellular environment (3). While the PEP–pyruvate–OAA node (4) could potentially create a futile cycle, this does not seem to contribute significantly to the physiological defects observed from pyruvate kinase dysregulation.

In this study, we identified a regulatory mechanism that prevents metabolic conflict during gluconeogenesis (Fig. 6). We found that in *B. subtilis* and related bacteria, pyruvate kinase is inhibited during gluconeogenesis via its ECTD regulatory domain. This inhibition blocks the conversion of PEP to pyruvate, thereby expanding the PEP pool, promoting efficient carbon utilization, reducing carbon overflow, and enabling faster growth. Conversely, mutants lacking this regulatory domain exhibit constitutively active pyruvate kinase in *B. subtilis*, triggering simultaneous glycolytic and gluconeogenic reactions during gluconeogenesis, compromising carbon usage and slowing growth. Moreover, this dysregulation renders bacteria more vulnerable to glyphosate, which interferes with the synthesis of aromatic amino acids from PEP. Due to the inability to sustain high PEP levels, the constitutive pyruvate kinase mutants are more sensitive to glyphosate. Thus, regulating pyruvate kinase is pivotal for metabolic coordination, significantly impacting carbon efficiency, growth optimization, and resistance to antimicrobial challenges.

Current knowledge on allosteric regulation of pyruvate kinase has been focused on glycolysis (7, 16). However, our research shows that dysregulation of pyruvate kinase leads to three defective phenotypes exclusively during gluconeogenesis linked to high PEP levels and carbon overflow. Our *in silico* modeling suggests that the thermodynamically unfavorable processes inherent in gluconeogenesis require high PEP levels sustained through pyruvate kinase regulation. We speculate that these elevated PEP levels play a crucial role in facilitating timely generation of glycolytic intermediates, enabling resource allocation to important cellular processes, such as nucleotide and cell envelope synthesis. In mammalian cells, constitutively active pyruvate kinase is proposed to decrease the glycolytic intermediates branching into biosynthetic pathways for nucleotides and some amino acids (37), although depletion of glycolytic intermediates is not observed in the mammalian metabolome during glycolytic growth (38). Using a bacterial model system, we demonstrate that while constitutively active pyruvate kinase does not affect glycolytic intermediates during glycolytic growth, it fails to support the accumulation of these intermediates during gluconeogenic growth, leading to defective anabolism. Thus, the role of pyruvate kinase regulation for proliferation may be a conserved mechanism across bacteria and humans.

While our study provided a comprehensive picture on how the lack of pyruvate kinase inhibition results in metabolic conflict, it remains unclear how ECTD enables the inhibition of pyruvate kinase activity during gluconeogenesis. Pyruvate kinases from many species have been shown to be allosterically regulated by multiple effectors (9–15, 39). The binding of allosteric effectors leads to the conformational change of the tetramer between inactive “T-state” and active “R-state” (13, 40, 41). The effector binding sites in pyruvate kinase are located on its C domain near the dimer–dimer interface instead of directly on the ECTD. Instead, ECTD interfaces with adjoining monomers in a crystal structure of tetrameric pyruvate kinase (17). Based on our observations that ΔECTD pyruvate kinase remains as a tetramer, it is most likely that the ECTD is crucial for keeping the pyruvate kinase tetramer at “T-state” while allowing activation by effectors. In addition to mediating allosteric regulation, residues on ECTD may also undergo phosphorylation (18, 22–25, 42). However, substitutions of potential phosphorylation sites to alanine did not restore the growth rate regardless of growth conditions (Fig. S6d). Therefore, while ECTD offers valuable insight into the impact of the pyruvate kinase regulation on bacterial metabolism, the specific mechanisms by which ECTD regulates pyruvate kinase remain poorly understood.

Although we show that ECTD serves an autoinhibitory function of pyruvate kinases from representative Bacillota species, including *B. subtilis, B. anthracis, L. monocytogenes, E. faecalis*, and *L. rhamnosus*, exceptions exist. It is reported that ECTD promotes pyruvate kinase activity in *S. aureus* and does not have significant impact on pyruvate kinase activity from *Geobacillus thermophilus* (14, 42), although the *in vivo* role of ECTD in these species has not been characterized. Furthermore, it should be noted that beyond the Bacillota phylum, pyruvate kinases from species such as *Escherichia coli* and humans lack the ECTD yet are still allosterically regulated (9, 11), whereas the pyruvate kinase from *Zymomonas mobilis*, also lacking ECTD, is not allosterically regulated (43). Given the conservation of ECTD across Bacillota and Cyanobacteriota, it is possible that the loss or gain of this domain occurred before the divergence of modern bacteria phyla. Pyruvate kinases with the ECTD may have evolved the domain into an autoinhibitory domain to facilitate gluconeogenesis, while the pyruvate kinases without the ECTD likely evolved alternative regulatory mechanisms during gluconeogenesis. Finally, pyruvate kinase has been proposed to interact with DNA replication enzymes (19) and cell division in the Gram-positive bacterium *B. subtilis* (44). How pyruvate kinase regulation interacts with these other processes remains to be experimentally elucidated.

In the absence of pyruvate kinase inhibition, we observed lower growth yield and slower growth rate in cells during gluconeogenesis, which can be due to an energy-wasting PEP–pyruvate–OAA futile cycle (7). However, these growth defects persisted even when the futile cycle was genetically disrupted, indicating that futile cycle is not the main cause of the defective gluconeogenesis upon pyruvate kinase dysregulation. Instead, the lower carbon use efficiency can be explained by an increased carbon overflow into the media. In addition, the slow growth and increased sensitivity to glyphosate, a PEP competitor, can be explained by failure to build an expanded PEP pool. A similar example was reported in glycolysis where depletion of the TCA cycle intermediates, instead of activation of the futile cycle (45), resulted in growth defect. It is important to emphasize that pyruvate kinase inhibition may still contribute to prevention of the futile cycle, and that other mechanisms may be in place to prevent the futile cycle in the absence of pyruvate kinase regulation. Nevertheless, our results showed that pyruvate kinase regulation is important for maintaining a high PEP levels to drive synthesis of central carbon metabolic intermediates. This enables efficient generation and strategic allocation of resources toward essential cellular processes while minimizing the diversion to the TCA cycle and overflow metabolism.

In addition to increasing steady state growth rates of bacterial culture, allosteric regulation of pyruvate kinase is also important for adaptation to nutrient shifts. During nutrient shifts, allosteric regulation and gene regulation are both critical for reducing lag and promoting adaptation (46–48). It has been shown that allosteric regulation of pyruvate kinase is important for growth transition in yeast (4), and we have observed a prolonged lag time in the constitute *pyk* mutant in *L. rhamnosus*, due to diauxic shift caused by the complex media composition (Fig. S7). In the case of *B. subtilis*, the soil bacterium uses both glucose and malate as primary carbon sources, necessitating a rapid switch between gluconeogenesis and glycolysis for competitive fitness. By regulating pyruvate kinase activity, the bacterium can maintain high enzyme levels while swiftly adjusting to alternative carbon sources. Further research on the mechanisms of allosteric regulation for central metabolic enzymes, such as pyruvate kinase, will enhance our understanding of bacterial metabolic adaptation and how it impacts bacterial fitness in different natural environments.

## MATERIALS AND METHODS

### Phylogenetic analysis

For the phylogenetic analysis of pyruvate kinases containing an ECTD, 118 bacterial reference genomes from the NCBI genome database were compared (Table S1). Pyruvate kinase sequences were found in 113 out of 118 reference genomes based on annotations using Biopython (49) and were aligned with MUSCLE in MEGA X (50) using default settings. 16S rRNA sequences from these 113 genomes were identified based on the annotations using Biopython and aligned with ClustalW in MEGA X using default settings. A phylogenetic tree was built with MEGA X using the maximum likelihood method. Data were plotted with R package ggtree (51).

### Plasmid and strain construction

All strains and plasmids used in this study are listed in Table 2 and Table 3.

**TABLE 2:** Strains used in this study

Strain name	Genotype	Reference
DK3287^*a*^	*B. subtilis* NCIB3610 Δ*zpdN* ΔSPβ ΔPBSX Δ*comI*	B.-E. Myagmarjav (52)
JDW3056	DK3287 *pyk*Δ*ectd*	This work
JDW3420	DK3287Δ*pyk*	This work
JDW3955	DK3287 *pyk*^H539A^	This work
JDW4597	DK3287 *pyk*^T537A^	This work
JDW4563	DK3287 *pycA∷Kan*	This work
JDW4564	JDW3056 *pycA∷Kan*	This work
JDW4500	DK3287 *pyk*Δ*ectd pyk* nt *A ins@463*	This work
JDW4328	DK3287 *amyE*::P_spank_-*pyk*	This work
JDW4330	JDW3056 *amyE*::P_spank_-*pyk*	This work
JDW4512	JDW3420 *amyE*::P_spank_-*pyk*	This work
VPL1029	*L. rhamnosus* GG	ATCC 53103
VPL4404	VPL1029 *pyk*Δ*ectd*	This work

^*a*^ Referred to as wild type in this paper.

**TABLE 3:** Plasmids used in this study

Plasmid name	Construct	Purpose	Reference
pJW269	pLIC-trPC-HA	Expression vector	L. Stols (53)
pJW422	pLIC-trPC-HA-*pyk*	Expression of *B. subtilis* pyruvate kinase	This work
pJW724	pLIC-trPC-HA-*pyk*Δ*ectd*	Expression of *B. subtilis* ΔECTD pyruvate kinase (residues 1–474)	This work
pJW878	pLIC-trPC-HA-Ban *pyk*	Expression of *B. anthracis* pyruvate kinase	This work
pJW879	pLIC-trPC-HA-Ban *pyk*Δ*ectd*	Expression of *B. anthracis* ΔECTD pyruvate kinase (residues 1–474)	This work
pJW880	pLIC-trPC-HA-Efa *pyk*	Expression of *E. faecalis* pyruvate kinase	This work
pJW881	pLIC-trPC-HA-Efa *pyk*Δ*ectd*	Expression of *E. faecalis* ΔECTD pyruvate kinase (residues 1–474)	This work
pJW882	pLIC-trPC-HA-Lmo *pyk*	Expression of *L. monocytogenes* pyruvate kinase	This work
pJW883	pLIC-trPC-HA-Lmo *pyk*Δ*ectd*	Expression of *L. monocytogenes* ΔECTD pyruvate kinase (residues 1–474)	This work
pJW557	pPB41 (54) without T7 promoter by the recombination template insertion site	CRISPR editing of genome	B. Anderson (20)
pJW693	pJW557 with *pyk*Δ*ectd* guide RNA and repair template	CRISPR recombineering of *B. subtilis pyk*Δ*ectd*	This work
pJW442	pJW299 (55) with 500 bp upstream and 500 bp downstream sequence of *B. subtilis pyk*	Engineering *B. subtilis* Δ*pyk*	This work
pJW721	pJW557 with *pyk*^H539A^ guide RNA and repair template	CRISPR recombineering of *B. subtilis pyk*^H539A^	This work
pJW870	pJW557 with *pyk^T^*^537A^ guide RNA and repair template	CRISPR recombineering of *B. subtilis pyk^T^*^539A^	This work
pJW811	pDR110 (from D. Rudner) *amyE*::P_spank_-*B. subtilis pyk*	Express *B. subtilis* pyruvate kinase at non-endogenous site	This work

Removal of the ECTD coding region in the chromosomal *pyk* gene in *B. subtilis* was done using CRISPR/Cas9 editing (54) to replace the *pyk* gene with the *pykΔectd* allele. First, we modified the general purpose CRISPR/Cas9 vector pPB41 (54) by removing a T7 promoter upstream the repair template inserting site, preventing expression of the repair template sequence from expressing in *E. coli*, giving rise to pJW557. Next, the guide RNAs (oJW3089 [aaacACAGAAGAAGGCGGTTTGACTAGCCATGCTGg] and oJW3090 [aaaacCAGCATGGCTAGTCAAACCGCCTTCTTCTGT]), and the repair template (300 bp upstream and 300 bp downstream of ECTD) are inserted into pJW557 using Golden Gate Assembly (New England Biolabs), resulting in plasmid pJW724. Finally, pJW724 was transformed into wild-type NCIB3610 strain (DK3287) and screened for *pyk*Δ*ectd* by Sanger sequencing of *pyk*, and by Illumina whole genome sequencing to verify that there were no second-site mutations.

For the deletion of pyruvate kinase, the plasmid pJW442 was constructed by inserting 500 bp upstream and downstream of *pyk* into pEX44 (56). pJW442 was then transformed into DK3287, followed by transformation of I-sceI expression vector pJW296 to induce recombination, and colonies of the Δ*pyk* mutant were purified and confirmed by Sanger sequencing of PCR product.

VPL4404 (*L. rhamnosus* GG *pyk*Δ*ectd* retaining the N-terminal residues 1–477) was constructed by homologous recombination using dipeptide ligase as the counterselection marker (57).

For the complementation experiment, *pyk* open reading frame was inserted into PDR110 by restriction enzyme digestion and ligation to get pJW811. pJW811 was transformed into DK3287 (wild type), JDW3056 (*pyk*Δ*ectd*), and JDW3420 (Δ*pyk*) to get JDW4328, JDW4330, and JDW4512 respectively.

### Protein expression and purification

For pyruvate kinase expression and purification, full-length pyruvate kinase or the ΔECTD variant (retaining the N-terminal residues 1–474 for *B. subtilis*, *B. anthracis, E. faecalis*, or *L. monocytogenes* pyruvate kinase) coding sequences were cloned into the pLIC-trPC-HA vector (53) (pJW269) downstream of a sequence encoding the hexa-histidine tag and the tobacco etch virus (TEV) protease cleavage site using ligation-independent cloning (LIC) (53). Expression vectors were transformed into *E. coli* BL21 (DE3) (NEB). A small-scale seed culture was grown in LB with 100 µg/mL carbenicillin to OD_600_ around 0.8 and sub-cultured 1:50 into fresh LB with 100 µg/mL carbenicillin. Then, 1 mM isopropyl β-D-1 thiogalactopyranoside (IPTG) (RPI) was added at around OD_600_ 0.8 to induce the expression. After 4 h of induction at 37°C, cells were pelleted and frozen at −80°C until purification.

For protein purification, cell pellets were suspended in lysis buffer (25 mM Tris-HCl [pH = 7.5], 300 mM NaCl, 10 mM imidazole) supplemented with 33 µg/mL deoxyribonuclease I (to reduce the viscosity of the cell lysate), followed by cell lysis using French press. The cell lysate was centrifuged at 12,000 × *g* 4°C for 30 min to obtain the supernatant, which is subsequently filtered before loading to a HisTrap FF Column (Cytiva) on AKTA pure FPLC (Cytiva). The column was washed with 34.5 mM imidazole in lysis buffer for five column volumes and eluted with an increasing gradient of imidazole from 34.5 to 500 mM for 15 column volumes. His-tagged enzymes were digested with TEV protease to remove the hexa-histidine tag, and further purified by size-exclusion chromatography. Enzymes were concentrated to ~8 mg/mL, and the enzyme concentration was measured by Bradford assay (Bio-Rad). Enzymes were stored in a buffer with 30 mM Tris-HCl (pH = 7.5), 100 mM NaCl, 5 mM MgCl_2_, 25 mM KCl, 1 mM DTT, and 10% (vol/vol) glycerol before being flash frozen with liquid nitrogen.

### Size exclusion chromatography

One hundred microliters of 100 µM wild-type or ΔECTD pyruvate kinase was applied onto a Superdex200 26/60 Gl column (GE Healthcare) and eluted with SEC buffer (20 mM Tris-Cl [pH 7.5], 300 mM NaCl, 1 mM EDTA, 1 mM DTT and 10% [vol/vol] glycerol) at 3 mL/min flow rate at 4°C. A standard curve for molecular mass determination was obtained using a mixture of thyroglobulin (669 kDa), ferritin (440 kDa), aldolase (158 kDa), conalbumin (75 kDa), ovalbumin (43 kDa), and RNase A (13.7 kDa). The partition coefficient (*K*_av_) was calculated as per *K*_av_ = (*V*_*e*_ *− V*_*o*_)/(*V*_*c*_ *− V*_*o*_) with the elution volumes (*V*_*e*_) of the peak apex of the respective proteins, the given void volume of the column (*V*_*o*_) of 100 mL and a total column volume (*V*_*c*_) of 300 mL.

### Pyruvate kinase kinetics assay

For all *in vitro* assays, pyruvate kinase activity was measured using an established coupled enzyme assay with pyruvate kinase and lactate dehydrogenase (21), in which the product of pyruvate kinase, pyruvate, is used by lactate dehydrogenase to convert NADH to NAD^+^. Production of NADH was then monitored by the loss of NADH absorbance at 340 nm.

The *B. subtilis* pyruvate kinase reaction mix contained 100 mM Tris-HCl (pH = 7.5), 100 mM KCl, 10 mM MgCl₂, 1.5 mM PEP (Millipore Sigma), 1 mM ADP (Millipore Sigma), 0.3 mM NADH (Millipore Sigma), and 25 U/mL L-lactate dehydrogenase (from bovine muscle, Millipore Sigma). When indicated, 1 mM AMP (Millipore Sigma) and/or 1 mM R5P (Biosynth International) and/or 5 mM ATP (Millipore Sigma) were added. Enzymatic reactions were initiated by addition of pyruvate kinase to a final concentration of 20 nM, and the absorbance at 340 nm was monitored in a quartz cuvette with a Shimadzu UV-2401PC spectrophotometer. Data were fitted using Python with Michaelis–Menten kinetics equations and plotted with Matplotlib (58).

Assay of *B. subtilis* pyruvate kinase activities at different combinations of substrate and activator concentrations was conducted in 96-well plates and the absorbance at 340 nm was monitored using a Synergy 2 microplate reader (BioTek). The reaction mix contained 100 mM Tris-HCl (pH = 7.5), 100 mM KCl, 10 mM MgCl₂, 0.5 mM ADP, 0.3 mM NADH, 25 U/mL L-lactate dehydrogenase, 5 mM ATP, and different concentrations of PEP (0, 0.2, 0.4, 0.6, 0.8, 1.0, 1.2, and 1.5 mM) and R5P (0, 20, 40, 60, 80, 100, 200, 300, 400, 500, 600, and 700 µM) and 4 nM pyruvate kinase to start the reaction. Data were analyzed using Python and plotted with Matplotlib (58). Wild-type pyruvate kinase activity was normalized to the ΔECTD pyruvate kinase activity based on the maximum velocity. Both wild-type and ΔECTD pyruvate kinase kinetics data were fit to a nonessential activation equation that Hill coefficient was adapted to (59):


$$v=\frac{V_{max}*{\left[S\right]}^{h}}{K_{S}*\frac{1+\frac{\left[A\right]}{K_{A}}}{1+\frac{\beta \left[A\right]}{\alpha K_{A}}}+{\left[S\right]}^{h}*\frac{1+\frac{\left[A\right]}{\alpha K_{A}}}{1+\frac{\beta \left[A\right]}{\alpha K_{A}}}}$$

where [*S*] represents substrate concentration, [*A*] represents activator concentration, and *h* represents the Hill coefficient. Data were fit to the equation above using SciPy (60).

Pyruvate kinases from other Bacillota species are tested with different concentrations of metabolic effectors for optimal activities. The *Bacillus anthracis* pyruvate kinase and DECTD variant reaction mix contained 1 mM AMP and 5 mM ATP. The *Enterococcus faecalis* pyruvate kinase and ΔECTD variant reaction mix contained 1 mM R5P. The *Listeria monocytogenes* pyruvate kinase and ΔECTD variant reaction mix contained 5 mM ATP.

### Biolog phenotypic screen

Wild-type and *pyk*Δ*ectd* mutant strains were streaked onto LB plates. Fresh colonies were used to inoculate cultures to an OD_600_ of 0.007 in inoculation fluid containing redox dye Dye Mix F (Biolog). Subsequently, 100 µL of the prepared culture was added to wells of PM1 and PM2 microplates (Biolog), each containing different carbon sources. Absorbance at 590 nm was measured using the OmniLog system for 24 h at 37°C. The data were analyzed with Python and visualized using Matplotlib (58).

### *B. subtilis* growth rate assay

To measure *B. subtilis* steady state growth rates, fresh colonies were used to inoculate cultures in a modified S7 defined media (61) [50 mM MOPS (pH adjusted to 7.0 with KOH), 10 mM (NH_4_)_2_SO_4_, 5 mM KH_2_PO_4_] (S7_50_ minimal media), supplemented with 1% (wt/vol) carbon source (i.e., glucose, malic acid [pH adjusted to 7 with KOH], sodium pyruvate), 200 µL culture in each well was grown in 96-well plates with vigorous shaking at 37°C, and optical density (OD_600_) was monitored by a Synergy 2 microplate reader (BioTek). Growth rate was obtained by fitting a linear regression model to the log-transformed OD values against time in the exponential phase. For maximum growth density assays, cells were washed with media without a carbon source, centrifuged, and resuspended into media with 0.2% (wt/vol) carbon source to an initial OD_600_ of 0.005. Then, 200 µL culture was added to each well in 96-well plates. Cells were grown with vigorous shaking at 37°C, and optical density (OD_600_) was monitored by a Synergy 2 microplate reader (BioTek).

For the complementation assay, cells were grown in the same way as above, except that 1 mM IPTG was added to the media to induce the expression of *pyk* when specified.

### *L. rhamnosus* GG growth assay

*L. rhamnosus* GG wild-type and *pyk*Δ*ectd* cells were inoculated in 5 mL De Man, Rogasa, and Sharpe (MRS; BD Difco) broth and incubated at 37°C for 16 h. Overnight cultures were washed twice with modified MRS (10 g peptone, 10 g beef extract, 5 g yeast extract, 2 g ammonium citrate dibasic, 0.1 g magnesium sulfate, 0.05 g manganese sulfate, 2 g dipotassium phosphate, and 1 mL Tween-80, dissolved in water to final volume 900 mL), followed by dilution into fresh mMRS supplemented with either 100 mM glucose, 100 mM of malate, or 100 mM of pyruvate to OD_600_ = 0.05. Growth was monitored by a plate reader (Multiskan Sky, Thermo Fisher) at 37°C under hypoxic conditions (5% CO_2_, 2% O_2_).

### Glyphosate resistance assays

For glyphosate resistance assays, cells were grown in the same way as the growth rate assay, except that 0, 0.094, 0.188, 0.375, 0.75, 1.5, 2, 2.5, 3, 4, and 5 mM of glyphosate were added to the media. Glyphosate stock solution was made by dissolving glyphosate [N-(phosphonomethyl) glycine] (Sigma-Aldrich) in 100 mM KOH to 100 mM.

### Glyphosate resistance suppressors

JDW4500 was obtained by plating JDW3056 on an agar plate made from S7_50_ minimal media supplemented with 1% (wt/vol) malic acid (pH adjusted to 7 with KOH), 1.5% (wt/vol) agar and 4.5 mM glyphosate. A single colony was obtained and verified. Genomic DNA was purified and sent for whole genome sequencing at SeqCenter. Mutation was identified from the sequencing result by breseq (62).

### Carbon use efficiency measurement

To measure carbon usage efficiency, cells were inoculated from single colonies on LB plates into S7_50_ minimal media supplemented with 1% (wt/vol) of the carbon source (glucose, pyruvate, or malate). Cultures were grown to an OD of ~0.5, washed with an equal volume of S7_50_ minimal medium without a carbon source, and resuspended in the same medium used for the pre-culture at an OD of ~0.005. Carbon usage was assessed by monitoring the remaining carbon source in the medium during exponential growth.

At each time point, 2 mL of culture was centrifuged at 16,100 × *g* for 1 min, and the supernatant was collected for quantification of the remaining carbon concentration. Additionally, 1 mL of culture was collected at each time point for OD measurement. Carbon sources in the media were quantified using different methods for glucose/pyruvate and malate, as described below. The remaining carbon concentrations in the medium were plotted against OD_600_, revealing a linear correlation. The slope of this correlation, representing the amount of carbon source consumed per unit of OD_600_, served as the indicator of carbon usage efficiency.

For quantification of glucose and pyruvate, media samples were mixed with 5 mM [U-¹³C] glucose or [1-¹³C] pyruvate as an internal control, and then quantified using LC-MS. MS parameters were set to a resolution of 140,000, an automatic gain control (AGC) of 1e6, a maximum injection time of 40 ms, and a scan range of 70–1,000 *m*/*z*. LC was performed on an ACQUITY UPLC BEH C18 column (1.7  µm, 2.1  ×  100  mm; Waters). Total run time was 15 min with a flow rate of 0.2 mL/min, using 97:3 (vol/vol) water/methanol, 10 mM tributylamine (pH 8.2–8.5 adjusted with ~9 mM acetic acid) as solvent A and 100% methanol as solvent B. The gradient was as follows: 0 min, 5% B; 2.5 min, 5% B; 7.5 min, 95% B; 9.5 min, 95% B; 10 min, 5% B; 15 min, 5% B.

For quantification of malate, media samples were acidified with 18.4 mM H_2_SO_4_. After 10 min, samples were centrifuged at 16,100 × *g* for 10 min, and the supernatant was loaded onto the HPLC and ran on a Phenomenex Rezex ROA-Organic Acid H^+^ column. Total run time was 45 min with a flow rate of 0.3 mL/min, using 7.6 mM H_2_SO_4_ as the mobile phase. Refractive index was monitored for the quantification, and concentrations were calculated based on a standard curve generated with 0.8, 4, and 20 mM malic acid standards.

### Metabolomic analysis by LC-MS

Cells were inoculated from single colonies into fresh media [50 mM MOPS (pH adjusted to 7.0 with KOH), 10 mM (NH_4_)_2_SO_4_, 5 mM KH_2_PO_4_, and 1% (wt/vol) carbon source; i.e., glucose, malic acid (pH adjusted to 7 with KOH), sodium pyruvate] and grown to OD_600_ of ~0.5. For sample collection, a 5 mL aliquot of culture was vacuum-filtered onto a 0.45 µm nylon membrane filter (Millipore), with three biological replicates obtained from parallel cultures. To rapidly quench metabolism, the membranes were immediately submerged in 1.5 mL of extraction solvent (acetonitrile:methanol:H_2_O = 40:40:20) pre-cooled on dry ice. The cell extracts were centrifuged at 13,200 × *g* for 10 min, and the supernatants were collected, combined with an internal control, dried under a nitrogen gas flow, and resuspended in HPLC-grade water.

The internal control consists of metabolites extracted from wild-type cells cultured in U-13C glucose. This control pool is then evenly distributed across samples to normalize the signals. For preparation of the internal control, wild-type cells were inoculated into media with 50 mM MOPS, 10 mM (NH_4_)_2_SO_4_, 5 mM KH_2_PO_4_, 1% (wt/vol) [U-¹³C] glucose, and grown at 37°C. Cells were inoculated into fresh [U-¹³C] glucose media in flasks at an initial OD_600_ ~0.05 and grown to OD_600_ of ~0.5. Metabolites were extracted as described above. Compounds with robust signals in the internal control were normalized to the internal control, while other compounds were normalized to the OD of the cell culture only, as detailed in the supplementary table.

For absolute metabolite concentration measurements, wild-type or *pyk*Δ*ectd* cells were inoculated into media with 50 mM MOPS, 10 mM (NH_4_)_2_SO_4_, 5 mM KH_2_PO_4_, and 1% (wt/vol) [U-¹³C] glucose or 1% (wt/vol) [1-^13^C] pyruvate or natural malate and grown in a 37°C shaker incubator overnight. Cells were inoculated into fresh corresponding media in flasks at an initial OD of ~0.05 and grown to OD of ~0.8. Unlabeled metabolites of interest were spiked in at 0×, 0.1×, 1×, and 10× of estimated concentrations into the extraction solvent before extraction for glucose and pyruvate samples. For the malate samples, 0× spiked samples from the pyruvate group were mixed in at a 1:1 ratio. Cell extracts were dried by nitrogen gas flow, and resuspended in HPLC grade H_2_O.

To detect metabolites, samples were analyzed using HPLC-MS consisting of a Dionex UHPLC coupled by electrospray ionization (ESI, negative mode) to a Q Exactive Orbitrap mass spectrometer (Thermo Scientific) operated in full-scan mode for detection of targeted compounds based on their accurate masses. MS parameters were set to a resolution of 70,000, an automatic gain control (AGC) of 1e6, a maximum injection time of 40 ms, and a scan range of 70–1,000 *m*/*z*. LC was performed on an ACQUITY UPLC BEH C18 column (1.7  µm, 2.1  ×  100  mm; Waters). Total run time was 25 min with a flow rate of 0.2 mL/min, using 97:3 (vol/vol) water/methanol, 10 mM tributylamine (pH 8.2–8.5 adjusted with ~9 mM acetic acid) as solvent A and 100% methanol as solvent B. The gradient was as follows: 0 min, 5% B; 2.5 min, 5% B; 17 min, 95% B; 19.5 min, 95% B; 20 min, 5% B; 25 min, 5% B. Raw output data from MS were converted to mzXML format using in-house-developed software, and quantification of metabolites was performed using Metabolomic Analysis and Visualization Engine (MAVEN) (63, 64). Ion counts of metabolites were normalized to OD_600_ and internal controls when applicable. If the ion counts of a metabolite in the internal control were too low, then the ion counts of this metabolite would be normalized to OD_600_ only.

*Z* scores were calculated within different strains and carbon sources using the formula *Z* = (*x* − µ)/σ, where *x* is normalized ion counts, µ is the average of normalized ion counts of a certain metabolite within different conditions, and σ is the standard error of normalized ion counts of a certain metabolite within different conditions.

### *In silico* methods for max–min driving force calculations

Optimized thermodynamic profiles for gluconeogenesis (2 pyruvate → glucose-6-phosphate) were generated using the max–min driving force (MDF) tool (36) via the Python package equilibrator pathway (version 0.5.0). Intracellular pH, ionic strength, and temperature were set to 7, 250 mM, and 298.15 K, respectively. Maximum and minimum concentration bounds for PEP, ATP, and ADP were based on a 40%, 100%, and 40% range of the calculated absolute intracellular concentrations (Table S2). The concentration bounds for the remaining metabolites were based on default ranges of 1 µM–10 mM. We chose a 100% concentration bound for ATP because ATP concentrations in *E. coli* have been found to vary more than 40% (65, 66). The concentration range of ATP in glucose-sufficient *E. coli* cells is shown to range between <1 to 8 mM, which is effectively a 100% variation assuming a mean of 4 mM. Other data show that many intracellular metabolite concentrations are relatively stable even across growth conditions (67).

To provide more conservative concentration ranges for the metabolites without absolute intracellular data for our *pyk*Δ*ectd* MDF profile, we combined the optimized metabolite concentrations generated from the wild-type model with our relative fold change data for the gluconeogenic metabolites and set a 40% range on those metabolite concentrations. Forward and net flux ratios for wild-type and mutant conditions were calculated using the least thermodynamically favorable free energy of the respective pathways (i.e., the MDF value) and the following equation: $\frac{J^{+}-J^{-}}{J^{+}+J^{-}}=\frac{e^{-\Delta G/RT}-1}{e^{-\Delta G/RT}+1}$ (36, 68).

## Data Availability

Source data for all figures and the metabolomics data have been deposited in Figshare at https://doi.org/10.6084/m9.figshare.27328287.v2.

## SUPPLEMENTAL MATERIAL

Supplemental Material msystems.01131-24-s0001.pdf Figures S1 to S11; Tables S1 to S3.

ASM does not own the copyrights to Supplemental Material that may be linked to, or accessed through, an article. The authors have granted ASM a non-exclusive, world-wide license to publish the Supplemental Material files. Please contact the corresponding author directly for reuse.
