# Supplementary material for: Allosteric regulation of pyruvate kinase enables efficient and robust gluconeogenesis by preventing metabolic conflicts and carbon overflow
Source: mSystems. 2025 Jan 28;10(2):e01131-24. doi: 10.1128/msystems.01131-24 (PMC11834443; doi:10.1128/msystems.01131-24)
Supplement: Supplemental Material — Figures S1 to S11; Tables S1 to S3. [file msystems.01131-24-s0001.pdf]

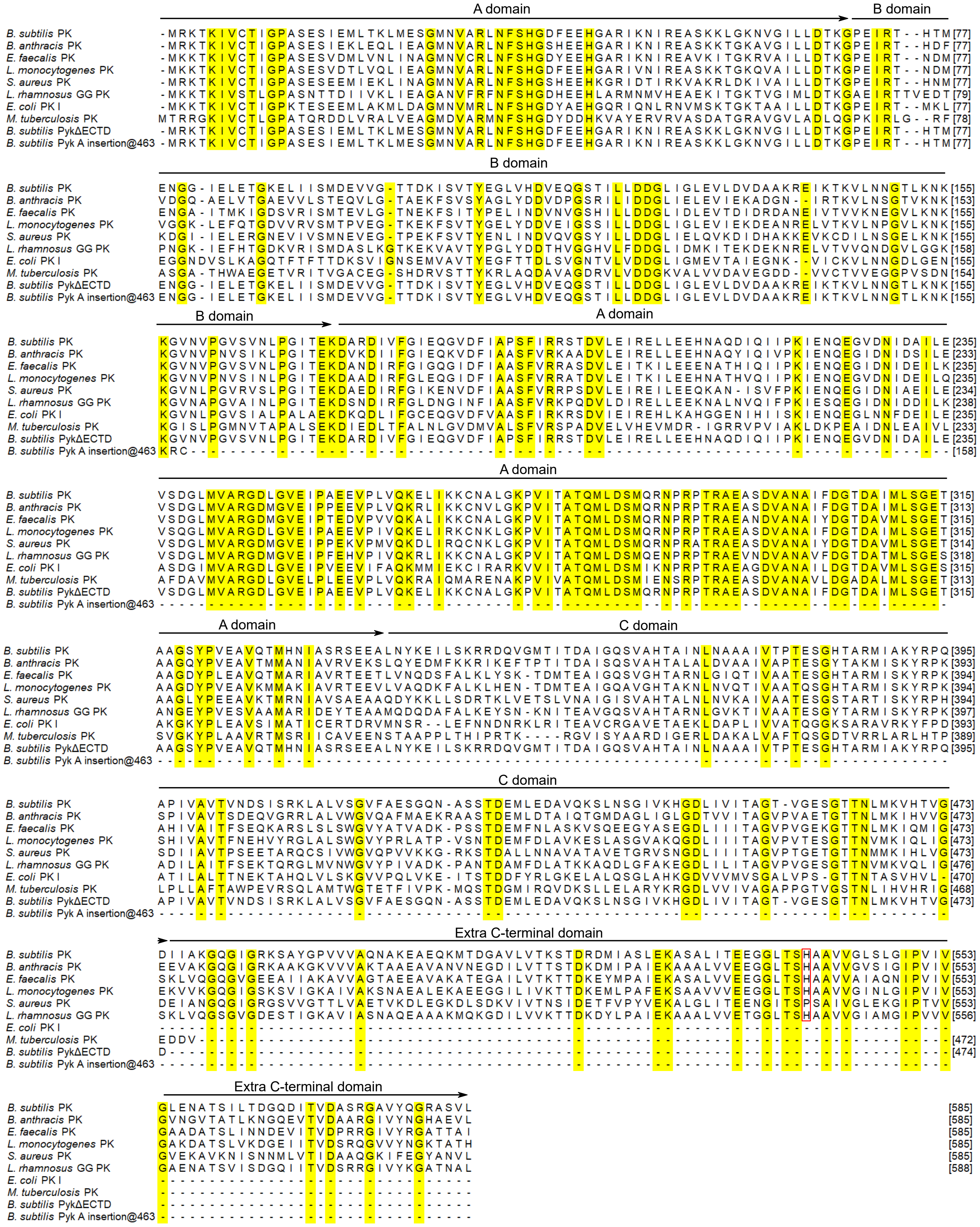

Supplementary Fig. 1: Pyruvate kinases in some bacteria contain an extra C-terminal domain.

Amino acid sequences alignment of pyruvate kinase in different species. Active site is in a pocket between A domain and B domain. C domain is a regulatory domain with effector binding sites. The active histidine on the extra C-terminal domain is circled by a rectangle, which is conserved in most Bacillota species but not *S. aureus*.

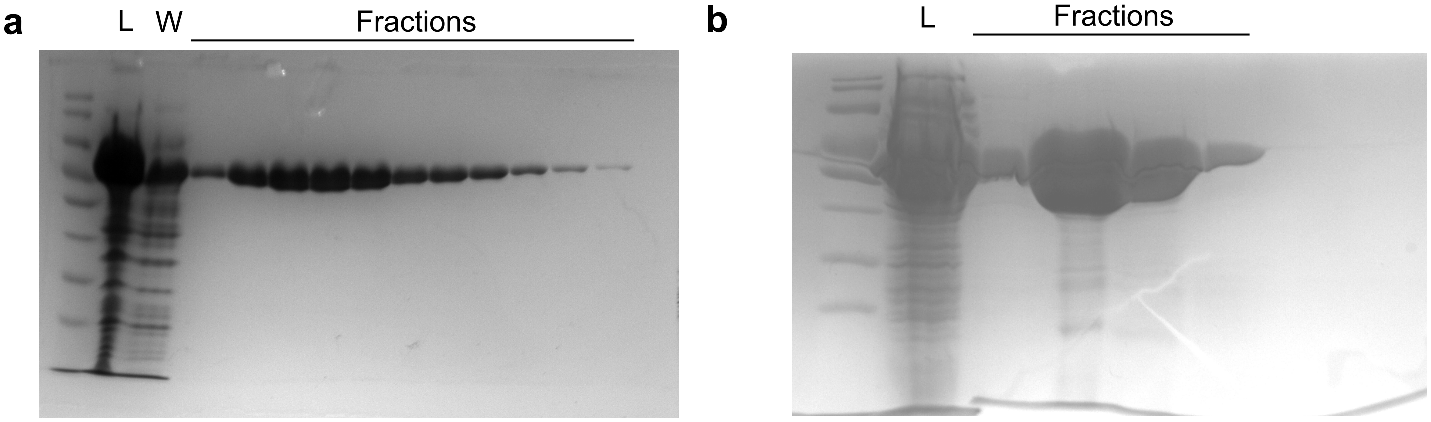

Supplementary Fig. 2: Purification of *B. subtilis* pyruvate kinases.
Coomassie brilliant blue stained SDS-PAGE gel of recombinantly expressed (a) wild type *Bacillus subtilis* pyruvate kinase and (b) ΔECTD *Bacillus subtilis* pyruvate kinase, purified with Ni-NTA column. L: lysate; W: wash.

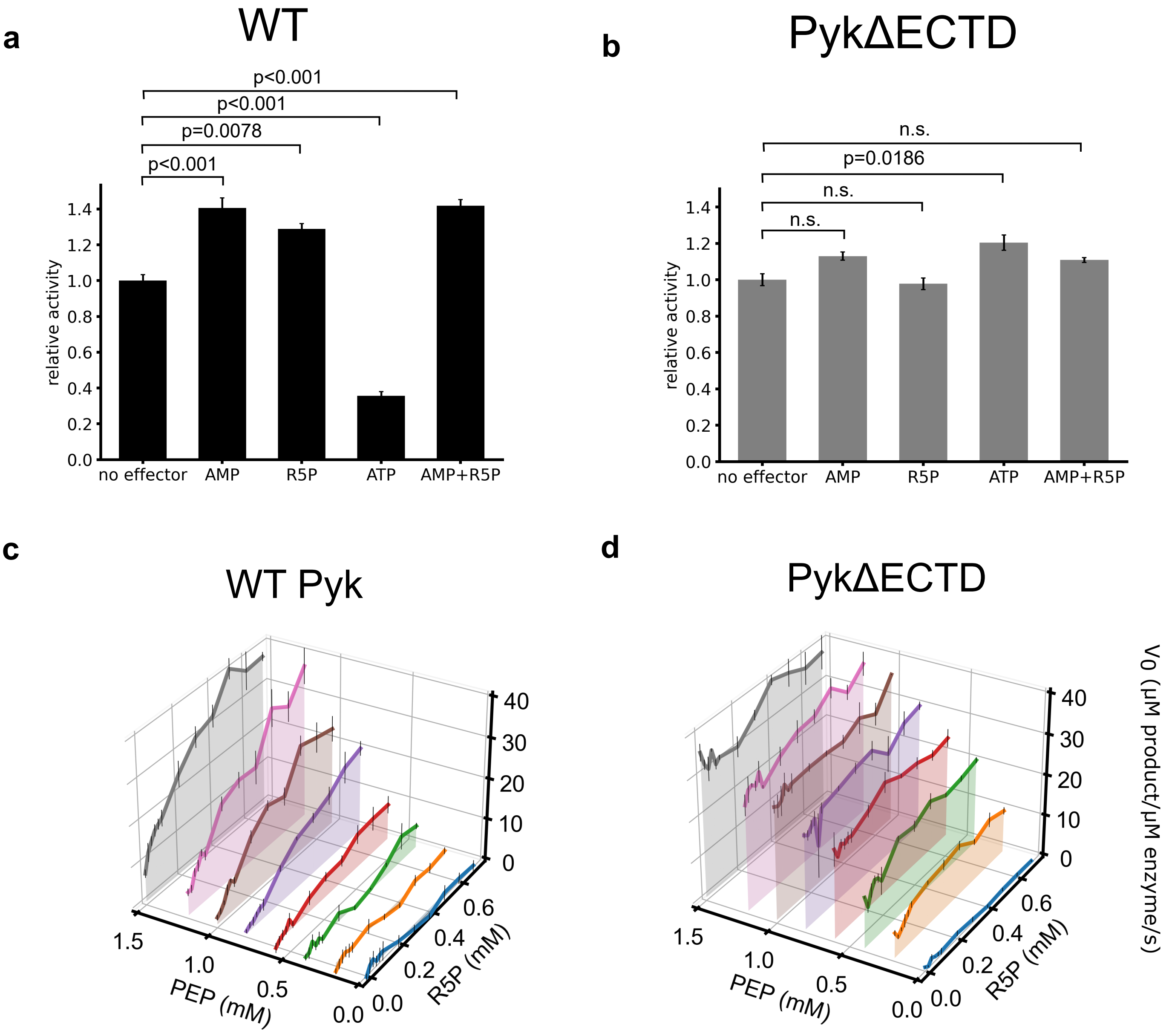

Supplementary Fig. 3: Three-dimensional analysis of pyruvate kinase kinetics: effects of substrate and activator concentrations on reaction rate.

(a)-(b) Regulation of allosteric effectors on (a) wild type enzyme and (b) the ΔECTD enzyme variant. (c)-(d) Pyruvate kinase activities with varying combination of the substate PEP and activator R5P from Fig. 1e and 1f.

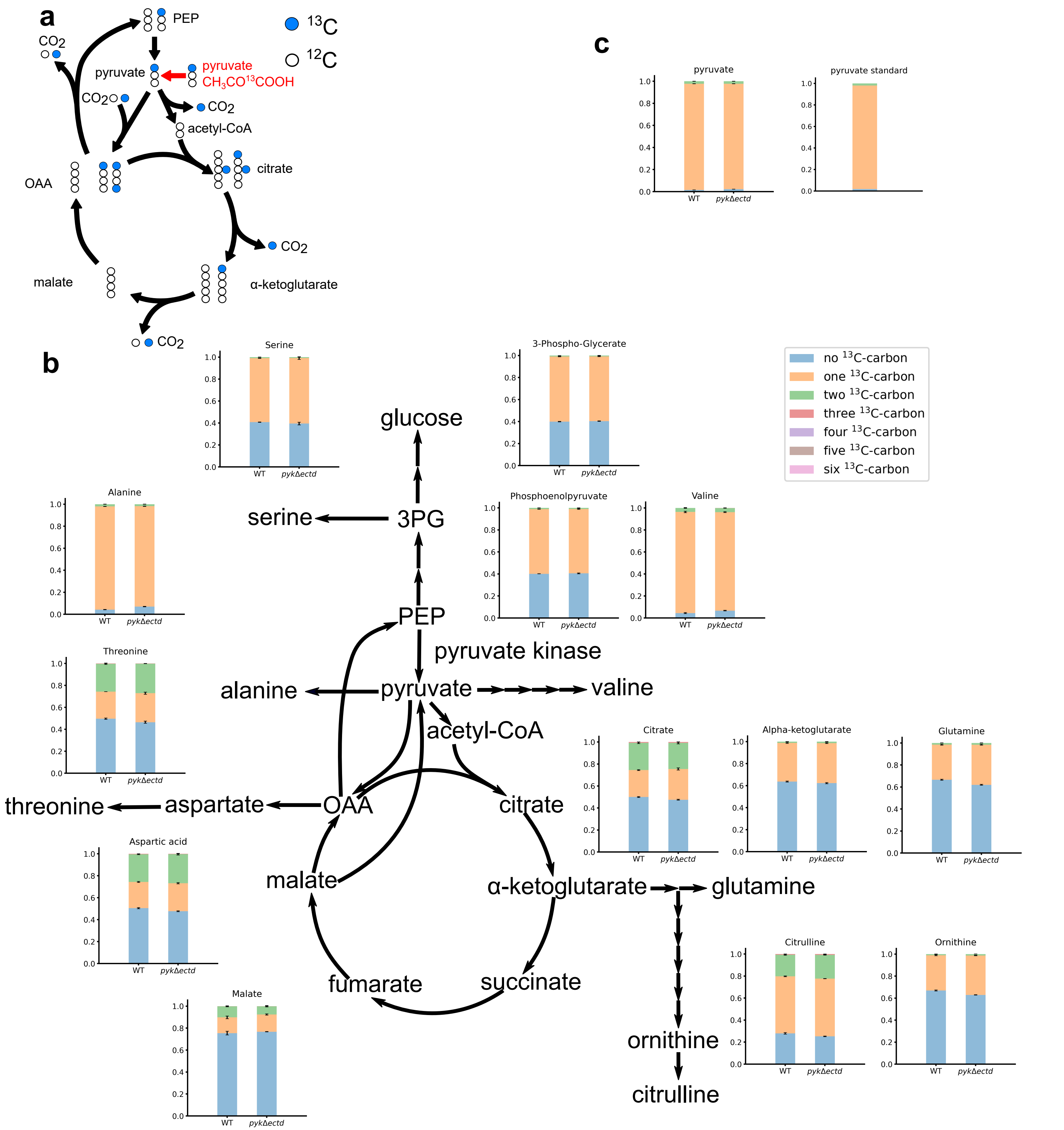

Supplementary Fig. 4: Isotope composition of important metabolites in cells grown in media with [1-^{13}C] pyruvate.(a) Schematics of the labeling experiment using [1-^{13}C] pyruvate in Fig. 2. In *B. subtilis*, PckA, which converts OAA to PEP, is the only enzyme that regenerates PEP from TCA cycle intermediates. Therefore, when pyruvate is the sole carbon source, all PEP is produced from OAA. OAA can be synthesized from pyruvate directly or from malate. When cells are grown on [1-^{13}C] pyruvate, OAA directly synthesized from pyruvate is [1-^{13}C] labeled. However, OAA synthesized from malate after one round of the TCA cycle is unlabeled. Therefore, only a fraction of OAA is expected to be labeled, as well as PEP since it is derived from OAA. (b) Wild type and *pykΔectd* cells were grown in media with [1-^{13}C] pyruvate as sole carbon source, isotope composition of intracellular metabolites was analyzed by LC-MS. Isotope composition of some metabolites in gluconeogenesis and TCA cycle, and some amino acids are shown here. (c) Isotope composition of pyruvate in cells (left) and in the media (right).

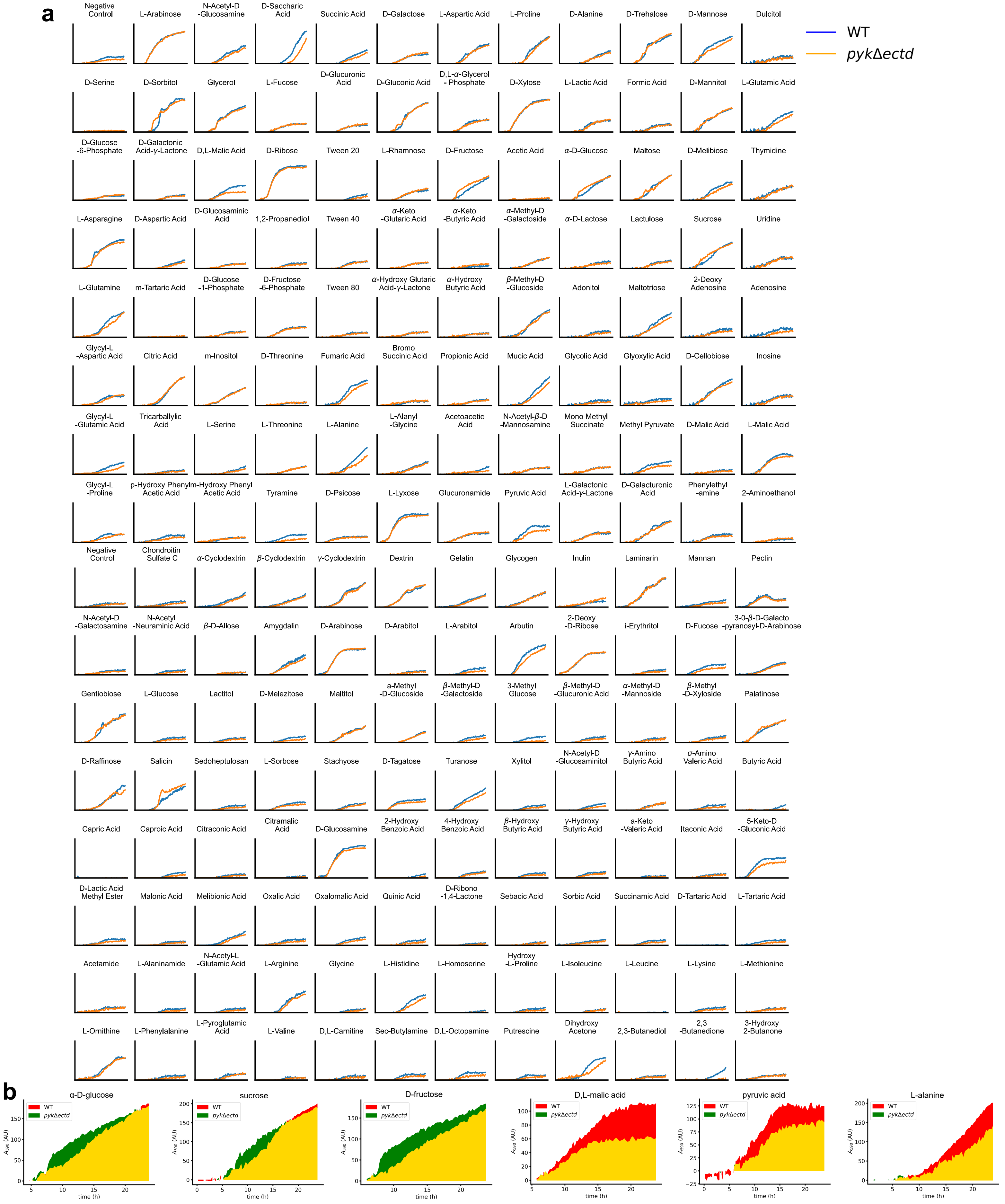

Supplementary Fig. 5: Phenotype profiling for *pykΔectd* mutant.

(a) Cells were inoculated in media with the indicated carbon sources and a redox dye which absorbs at 590 nm when reduced by cellular NADH as a reporter of growth. (b) Examples of several glycolytic (glucose, sucrose and fructose) and gluconeogenic carbon sources (malate, pyruvate and alanine) were plotted separately. Area under wild type curve is colored red, area under *pykΔectd* curve is colored green, overlapping region is colored yellow.

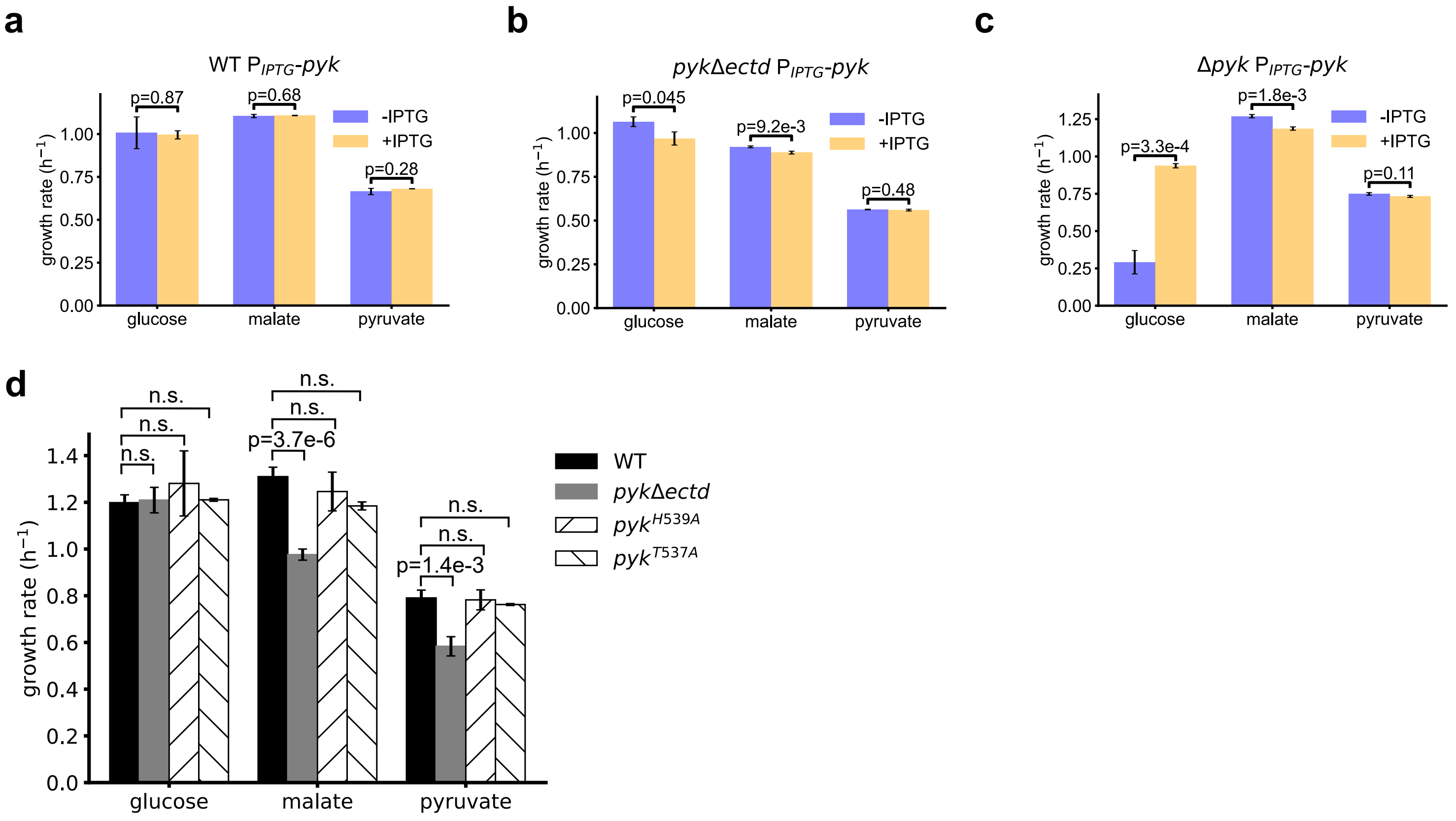

Supplementary Fig. 6: Complementation tests of *pyk* mutant alleles.

Growth rates of wild type (a), the *pyk*Δ*ectd* mutant (b), or the Δ*pyk* (c) mutant alleles complemented with wild type *pyk* growing in glycolytic (glucose) or gluconeogenic (malate or pyruvate) media. Wild type *pyk* was provided ectopically at the *amyE* locus with an IPTG-inducible promoter. (d) Growth rate of wild type, *pyk*Δ*ectd*, *pyk*^{H539A} and *pyk*^{T537A} in media with different sole carbon sources.

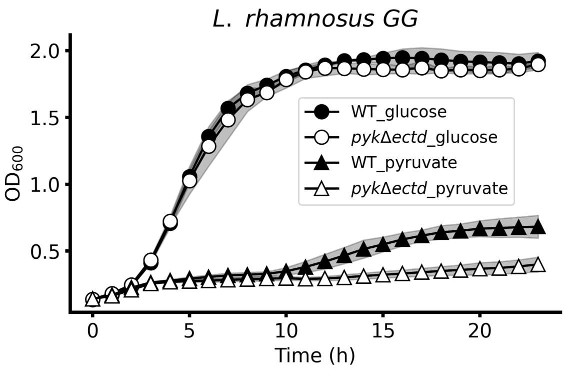

Supplementary Fig. 7: Growth curves of *L. rhamnosus GG* in media with glycolytic or gluconeogenic carbon sources.
Growth curve of the *L. rhamnosus GG* wild type and *pyk*Δ*ectd* cells in media with glucose or pyruvate as the sole carbon source. The same data as Fig. 2i.

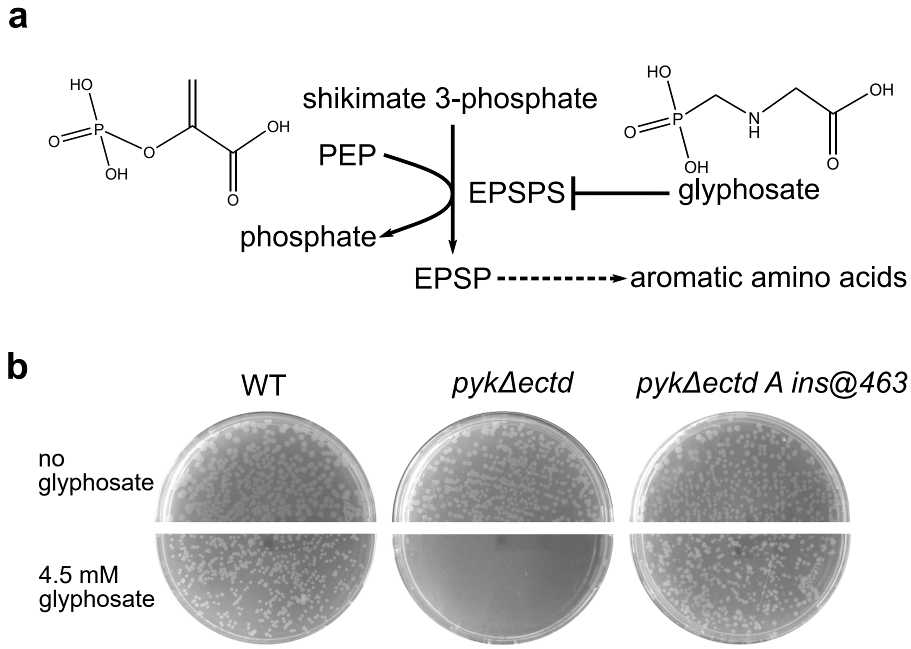

Supplementary Fig. 8: Hypersensitivity of *pyk*Δectd mutant to glyphosate can be suppressed by a loss-of-function mutation of *pyk*.
(a) Glyphosate inhibits the shikimate pathway which is required for the synthesis of aromatic amino acids by competing with PEP. EPSP: 5-enolpyruvylshikimate 3-phosphate; EPSPS: EPSP synthase. (b) Plate images of wild type, *pyk*Δ*ectd*, and glyphosate suppressor on tmalate minimal plates with or without glyphosate. The same data as Fig. 2l.

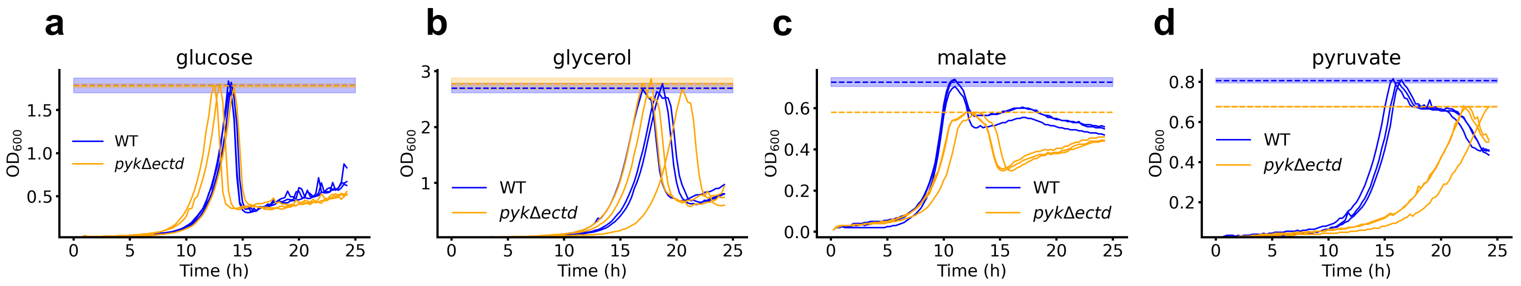

Supplementary Fig. 9: Maximum OD_{600} of wild type or mutant cells grown in media with different sole carbon sources. Growth curve of wild type or *pyk*Δ*ectd* cells in media with 2 g/L (a) glucose, (b) glycerol, (c) malate or (d) pyruvate as the sole carbon source, dash lines represent the average maximum ODs for each condition, colored region represents 95% confidence interval.

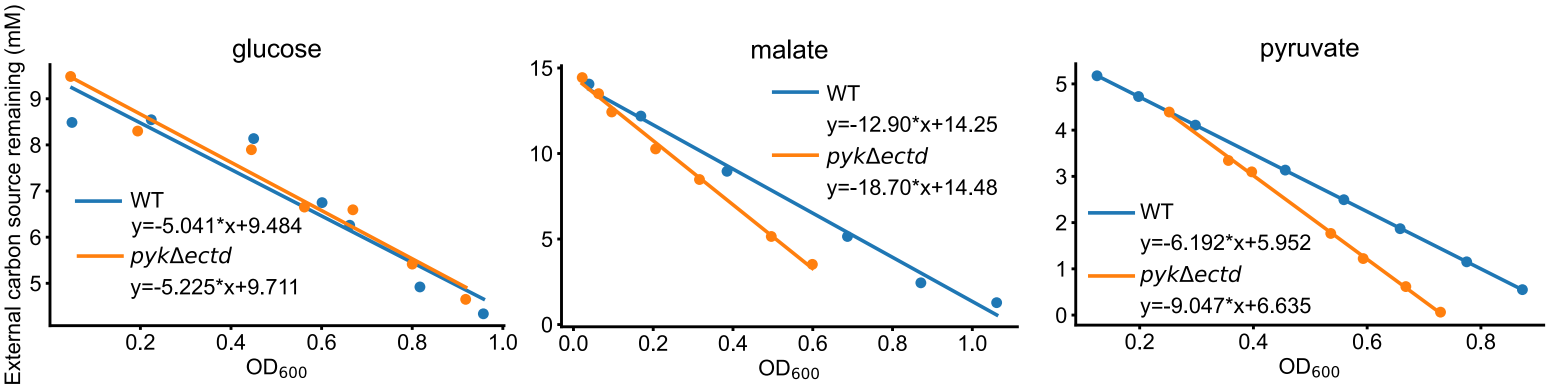

Supplementary Fig. 10: Gluconeogenic carbon use efficiency of *pyk*Δectd mutant is lower than wild type strain.

Carbon sources remaining in spent media plotted versus OD_{600} of cells. Slopes are calculated by linear regression with the method of least squares, which represents carbon use efficiency. One repeat for each carbon source is shown here.

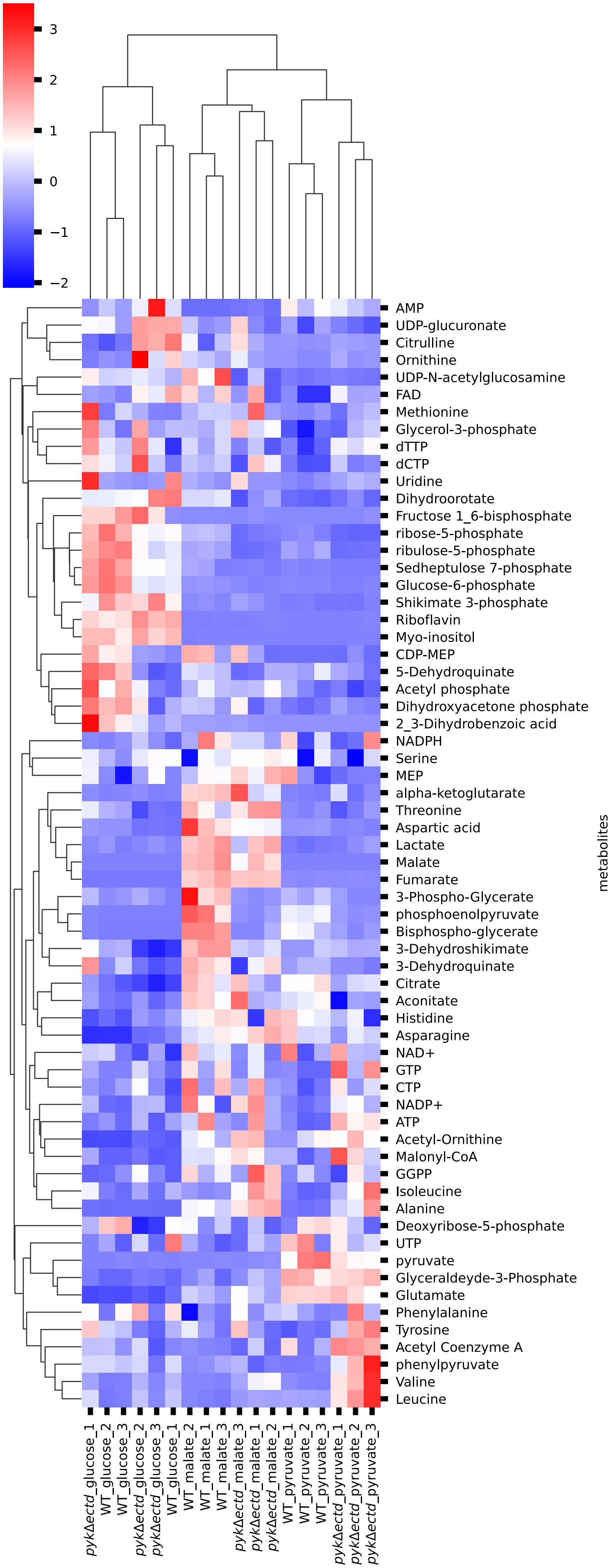

Supplementary Fig. 11: Metabolome of wild type and *pykΔectd* mutant grown in glycolytic or gluconeogenic media.

Heatmap of metabolites of wild type and *pykΔectd* cells grown in media with different carbon sources. Rows and columns are clustered with UPGMA (unweighted pair group method with arithmetic mean). Color represents Z scores for rows. MEP: 2-C-methyl-D-erythritol 4-phosphate; GGPP: geranylgeranyl pyrophosphate.

Supplementary Table 1: Distribution of pyruvate kinase ECTD in the 118 bacterial reference genomes

Accession	taxonomy	with pyk ECTD?
U00096	Bacteria_Proteobacteria_Gammaproteobacteria_Enterobacterales_Enterobacteriaceae_Escherichia	no
AE003852	Bacteria_Proteobacteria_Gammaproteobacteria_Vibrionales_Vibrionaceae_Vibrio	no
AE004091	Bacteria_Proteobacteria_Gammaproteobacteria_Pseudomonadales_Pseudomonadaceae_Pseudomonas	no
AE004092	Bacteria_Firmicutes_Bacilli_Lactobacillales_Streptococcaceae_Streptococcus	no
AE004969	Bacteria_Proteobacteria_Betaproteobacteria_Neisseriales_Neisseriaceae_Neisseria	no
AE005176	Bacteria_Firmicutes_Bacilli_Lactobacillales_Streptococcaceae_Lactococcus	no
AE005673	Bacteria_Proteobacteria_Alphaproteobacteria_Caulobacterales_Caulobacteraceae_Caulobacter	no
AE005674	Bacteria_Proteobacteria_Gammaproteobacteria_Enterobacterales_Enterobacteriaceae_Shigella	no
AE006468	Bacteria_Proteobacteria_Gammaproteobacteria_Enterobacterales_Enterobacteriaceae_Salmonella	no
AL591688	Bacteria_Proteobacteria_Alphaproteobacteria_Rhizobiales_Rhizobiaceae_Sinorhizobium/Ensifer group_Sinorhizobium	no
AE006470	Bacteria_Chlorobi_Chlorobia_Chlorobiales_Chlorobiaceae_Chlorobaculum	no pyk
AE007317	Bacteria_Firmicutes_Bacilli_Lactobacillales_Streptococcaceae_Streptococcus	no
AE008922	Bacteria_Proteobacteria_Gammaproteobacteria_Xanthomonadales_Xanthomonadaceae_Xanthomonas	no
AE009948	Bacteria_Firmicutes_Bacilli_Lactobacillales_Streptococcaceae_Streptococcus	no
AE009951	Bacteria_Fusobacteria_Fusobacteriales_Fusobacteriaceae_Fusobacterium	no
AE014133	Bacteria_Firmicutes_Bacilli_Lactobacillales_Streptococcaceae_Streptococcus	no
AE014295	Bacteria_Actinobacteria_Bifidobacteriales_Bifidobacteriaceae_Bifidobacterium	no
AE015451	Bacteria_Proteobacteria_Gammaproteobacteria_Pseudomonadales_Pseudomonadaceae_Pseudomonas	no
AE015929	Bacteria_Firmicutes_Bacilli_Bacillales_Staphylococcaceae_Staphylococcus	with pyk ECTD
AE016828	Bacteria_Proteobacteria_Gammaproteobacteria_Legionellales_Coxiellaceae_Coxiella	no
AE016830	Bacteria_Firmicutes_Bacilli_Lactobacillales_Enterococcaceae_Enterococcus	with pyk ECTD
AE016853	Bacteria_Proteobacteria_Gammaproteobacteria_Pseudomonadales_Pseudomonadaceae_Pseudomonas	no
AE016877	Bacteria_Firmicutes_Bacilli_Bacillales_Bacillaceae_Bacillus_Bacillus cereus group	with pyk ECTD
AE016879	Bacteria_Firmicutes_Bacilli_Bacillales_Bacillaceae_Bacillus_Bacillus cereus group	with pyk ECTD
AE017126	Bacteria_Cyanobacteria_Synechococcales_Prochloraceae_Prochlorococcus	with pyk ECTD
AE017180	Bacteria_Proteobacteria_Deltaproteobacteria_Desulfuromonadales_Geobacteraceae_Geobacter	no
AE017225	Bacteria_Firmicutes_Bacilli_Bacillales_Bacillaceae_Bacillus_Bacillus cereus group	with pyk ECTD
AE017226	Bacteria_Spirochaetes_Spirochaetales_Spirochaetaceae_Treponema	no
AE017263	Bacteria_Tenericutes_Mollicutes_Entomoplasmatales_Entomoplasmataceae_Mesoplasma	no
AE017354	Bacteria_Proteobacteria_Gammaproteobacteria_Legionellales_Legionellaceae_Legionella	no
AE017355	Bacteria_Firmicutes_Bacilli_Bacillales_Bacillaceae_Bacillus_Bacillus cereus group	with pyk ECTD
AE000511	Bacteria_Proteobacteria_Epsilonproteobacteria_Campylobacterales_Helicobacteraceae_Helicobacter	no pyk
AE000512	Bacteria_Thermotogae_Thermotogales_Thermotogaceae_Thermotoga	no
AE000513	Bacteria_Deinococcus-Thermus_Deinococci_Deinococcales_Deinococcaceae_Deinococcus	no
AE000657	Bacteria_Aquificae_Aquificales_Aquificaceae_Aquifex	no pyk
AE000783	Bacteria_Spirochaetes_Spirochaetales_Borreliaceae_Borreliella	no
AE001273	Bacteria_Chlamydiae_Chlamydiales_Chlamydiaceae_Chlamydia/Chlamydophila group_Chlamydia	no
AE001363	Bacteria_Chlamydiae_Chlamydiales_Chlamydiaceae_Chlamydia/Chlamydophila group_Chlamydia	no
AE001437	Bacteria_Firmicutes_Clostridia_Clostridiales_Clostridiaceae_Clostridium	no
AE002098	Bacteria_Proteobacteria_Betaproteobacteria_Neisseriales_Neisseriaceae_Neisseria	no
BA000007	Bacteria_Proteobacteria_Gammaproteobacteria_Enterobacterales_Enterobacteriaceae_Escherichia	no
CP000233	Bacteria_Firmicutes_Bacilli_Lactobacillales_Lactobacillaceae_Lactobacillus	with pyk ECTD
AJ749949	Bacteria_Proteobacteria_Gammaproteobacteria_Thiotrichales_Francisellaceae_Francisella	no
AL009126	Bacteria_Firmicutes_Bacilli_Bacillales_Bacillaceae_Bacillus	with pyk ECTD
AL590842	Bacteria_Proteobacteria_Gammaproteobacteria_Enterobacterales_Yersiniaceae_Yersinia	no
AL111168	Bacteria_Proteobacteria_Epsilonproteobacteria_Campylobacterales_Campylobacteraceae_Campylobacter	no
AM180355	Bacteria_Firmicutes_Clostridia_Clostridiales_Peptostreptococcaceae_Clostridioides	with pyk ECTD
AM286415	Bacteria_Proteobacteria_Gammaproteobacteria_Enterobacterales_Yersiniaceae_Yersinia	no
BA000003	Bacteria_Proteobacteria_Gammaproteobacteria_Enterobacterales_Erwiniaceae_Buchnera	no
AP006841	Bacteria_Bacteroidetes_Bacteroidia_Bacteroidales_Bacteroidaceae_Bacteroides	no
AE015928	Bacteria_Bacteroidetes_Bacteroidia_Bacteroidales_Bacteroidaceae_Bacteroides	no
BA000036	Bacteria_Actinobacteria_Corynebacteriales_Corynebacteriaceae_Corynebacterium	no
BA000039	Bacteria_Cyanobacteria_Synechococcales_Synechococcaceae_Thermosynechococcus	with pyk ECTD
BA000040	Bacteria_Proteobacteria_Alphaproteobacteria_Rhizobiales_Bradyrhizobiaceae_Bradyrhizobium	no
BA000045	Bacteria_Cyanobacteria_Gloeobacteria_Gloeobacterales_Gloeobacteraceae_Gloeobacter	with pyk ECTD
BX293980	Bacteria_Tenericutes_Mollicutes_Mycoplasmataceae_Mycoplasma	no
BX571965	Bacteria_Proteobacteria_Betaproteobacteria_Burkholderiales_Burkholderiaceae_Burkholderia_pseudomallei group	no
CP000010	Bacteria_Proteobacteria_Betaproteobacteria_Burkholderiales_Burkholderiaceae_Burkholderia_pseudomallei group	no
CP000020	Bacteria_Proteobacteria_Gammaproteobacteria_Vibrionales_Vibrionaceae_Aliivibrio	no
CP000033	Bacteria_Firmicutes_Bacilli_Lactobacillales_Lactobacillaceae_Lactobacillus	with pyk ECTD
CP000034	Bacteria_Proteobacteria_Gammaproteobacteria_Enterobacterales_Enterobacteriaceae_Shigella	no
CP000075	Bacteria_Proteobacteria_Gammaproteobacteria_Pseudomonadales_Pseudomonadaceae_Pseudomonas_Pseudomonas syringae	no
CP000159	Bacteria_Bacteroidetes_Bacteroidetes Order II. Incertae sedis_Rhodothermaceae_Salinibacter	no
CP000230	Bacteria_Proteobacteria_Alphaproteobacteria_Rhodospirillales_Rhodospirillaceae_Rhodospirillum	no
CP000232	Bacteria_Firmicutes_Clostridia_Thermoanaerobacterales_Thermoanaerobacteraceae_Moorella group_Moorella	with pyk ECTD
CP000253	Bacteria_Firmicutes_Bacilli_Bacillales_Staphylococcaceae_Staphylococcus	with pyk ECTD
CP000387	Bacteria_Firmicutes_Bacilli_Lactobacillales_Streptococcaceae_Streptococcus	no
CP000423	Bacteria_Firmicutes_Bacilli_Lactobacillales_Lactobacillaceae_Lactobacillus	with pyk ECTD
CP000462	Bacteria_Proteobacteria_Gammaproteobacteria_Aeromonadales_Aeromonadaceae_Aeromonas	no
CP000480	Bacteria_Actinobacteria_Corynebacteriales_Mycobacteriaceae_Mycobacterium	no
CP000727	Bacteria_Firmicutes_Clostridia_Clostridiales_Clostridiaceae_Clostridium	with pyk ECTD
CP000738	Bacteria_Proteobacteria_Alphaproteobacteria_Rhizobiales_Rhizobiaceae_Sinorhizobium/Ensifer group_Sinorhizobium	no
CP001389	Bacteria_Proteobacteria_Alphaproteobacteria_Rhizobiales_Rhizobiaceae_Sinorhizobium/Ensifer group_Sinorhizobium	no
CP000909	Bacteria_Chloroflexi_Chloroflexia_Chloroflexales_Chloroflexineae_Chloroflexaceae_Chloroflexus	no
CP001147	Bacteria_Nitrospirae_Nitrospirales_Nitrospiraceae_Thermodesulfovibrio	no
CP001251	Bacteria_Dictyoglomi_Dictyoglomales_Dictyoglomaceae_Dictyoglomus	with pyk ECTD

CP001340	Bacteria_Proteobacteria_Alphaproteobacteria_Caulobacterales_Caulobacteraceae_Caulobacter	no
CP001643	Bacteria_Actinobacteria_Micrococcales_Dermabacteraceae_Brachybacterium	no
CP001818	Bacteria_Synergistetes_Synergistia_Synergistales_Synergistaceae_Thermanaerovibrio	with pyk ECTD
CP001918	Bacteria_Proteobacteria_Gammaproteobacteria_Enterobacterales_Enterobacteriaceae_Enterobacter_Enterobacter cloacae complex	no
CU928164	Bacteria_Proteobacteria_Gammaproteobacteria_Enterobacterales_Enterobacteriaceae_Escherichia	no
FM252032	Bacteria_Firmicutes_Bacilli_Lactobacillales_Streptococcaceae_Streptococcus	no
FN568063	Bacteria_Firmicutes_Bacilli_Lactobacillales_Streptococcaceae_Streptococcus	no
L42023	Bacteria_Proteobacteria_Gammaproteobacteria_Pasteurellales_Pasteurellaceae_Haemophilus	no
U00089	Bacteria_Tenericutes_Mollicutes_Mycoplasmataceae_Mycoplasma	no
AM412317	Bacteria_Firmicutes_Clostridia_Clostridiales_Clostridiaceae_Clostridium	with pyk ECTD
AM398681	Bacteria_Bacteroidetes_Flavobacteriia_Flavobacteriales_Flavobacteriaceae_Flavobacterium	no
AM884176	Bacteria_Chlamydiae_Chlamydiales_Chlamydiaceae_Chlamydia/Chlamydophila group_Chlamydia	no
CU458896	Bacteria_Actinobacteria_Corynebacteriales_Mycobacteriaceae_Mycobacteroides abscessus	no
AP008226	Bacteria_Deinococcus-Thermus_Deinococci_Thermales_Thermaceae_Thermus	no
AE007869	Bacteria_Proteobacteria_Alphaproteobacteria_Rhizobiales_Rhizobiaceae_Rhizobium/Agrobacterium group_Agrobacterium_Agrobacterium tumefaciens complex	no
AE010300	Bacteria_Spirochaetes_Leptospirales_Leptospiraceae_Leptospira	no
AE014299	Bacteria_Proteobacteria_Gammaproteobacteria_Alteromonadales_Shewanellaceae_Shewanella	no
CP002104	Bacteria_Actinobacteria_Bifidobacteriales_Bifidobacteriaceae_Gardnerella	no
CP001840	Bacteria_Actinobacteria_Bifidobacteriales_Bifidobacteriaceae_Bifidobacterium	no
CP003583	Bacteria_Firmicutes_Bacilli_Lactobacillales_Enterococcaceae_Enterococcus	with pyk ECTD
CP001855	Bacteria_Proteobacteria_Gammaproteobacteria_Enterobacterales_Enterobacteriaceae_Escherichia	no
CP002447	Bacteria_Proteobacteria_Alphaproteobacteria_Rhizobiales_Phyllobacteriaceae_Mesorhizobium	no
CP002177	Bacteria_Proteobacteria_Gammaproteobacteria_Pseudomonadales_Moraxellaceae_Acinetobacter_Acinetobacter calcoaceticus/baumannii complex	no pyk
BX470248	Bacteria_Proteobacteria_Betaproteobacteria_Burkholderiales_Alcaligenaceae_Bordetella	no
AJ235269	Bacteria_Proteobacteria_Alphaproteobacteria_Rickettsiales_Rickettsiaceae_Rickettsieae_Rickettsia_typhus group	no pyk
AE017285	Bacteria_Proteobacteria_Deltaproteobacteria_Desulfovibrionales_Desulfovibrionaceae_Desulfovibrio	no
LT708304	Bacteria_Actinobacteria_Corynebacteriales_Mycobacteriaceae_Mycobacterium_Mycobacterium tuberculosis complex	no
AL450380	Bacteria_Actinobacteria_Corynebacteriales_Mycobacteriaceae_Mycobacterium	no
AL123456	Bacteria_Actinobacteria_Corynebacteriales_Mycobacteriaceae_Mycobacterium_Mycobacterium tuberculosis complex	no
AL513382	Bacteria_Proteobacteria_Gammaproteobacteria_Enterobacterales_Enterobacteriaceae_Salmonella	no
AL591824	Bacteria_Firmicutes_Bacilli_Bacillales_Listeriaceae_Listeria	with pyk ECTD
BA000031	Bacteria_Proteobacteria_Gammaproteobacteria_Vibrionales_Vibrionaceae_Vibrio	no
BX119912	Bacteria_Planctomycetes_Planctomycetia_Planctomycetales_Planctomycetaceae_Rhodopirellula	no
CP002000	Bacteria_Actinobacteria_Pseudonocardiales_Pseudonocardiaceae_Amycolatopsis	no
AL645882	Bacteria_Actinobacteria_Streptomycetales_Streptomycetaceae_Streptomyces_Streptomyces albidoflavus group	no
AL935263	Bacteria_Firmicutes_Bacilli_Lactobacillales_Lactobacillaceae_Lactobacillus	with pyk ECTD
CP002824	Bacteria_Proteobacteria_Gammaproteobacteria_Enterobacterales_Enterobacteriaceae_Klebsiella	no
CP002018	Bacteria_Proteobacteria_Alphaproteobacteria_Rhodobacterales_Rhodobacteraceae_Ketogulonicigenium	no
CP003200	Bacteria_Proteobacteria_Gammaproteobacteria_Enterobacterales_Enterobacteriaceae_Klebsiella	no
CP003289	Bacteria_Proteobacteria_Gammaproteobacteria_Enterobacterales_Enterobacteriaceae_Escherichia	no
HE965803	Bacteria_Proteobacteria_Betaproteobacteria_Burkholderiales_Alcaligenaceae_Bordetella	no
HE965806	Bacteria_Proteobacteria_Betaproteobacteria_Burkholderiales_Alcaligenaceae_Bordetella	no

Supplementary Table 2: Absolute concentration of some metabolites in wild type and *pyk* Δ*ectd* cells grown in different carbon sources (Unit: mM)

	Glucose		Malate		Pyruvate	
	Wild type	*pyk* Δ*ectd*	Wild type	*pyk* Δ*ectd*	Wild type	*pyk* Δ*ectd*
PEP	0.11±0.0040*	0.10±0.010	3.6±0.29	1.2±0.16	3.3±0.35	0.75±0.040
R5P	0.68±0.0079	0.73±0.038	0.30±0.016	0.12±0.024	0.23±0.070	0.05±0.020
ATP	3.1±0.17	3.2±0.042	4.4±0.56	4.8±0.63	4.1±0.55	3.6±0.32
ADP	0.66±0.066	0.78±0.027	1.4±0.035	1.3±0.032	0.95±0.20	0.45±0.063
AMP	0.12±0.025	0.12±0.025	ND[2]	ND[2]	0.20±0.12	0.15±0.17

* Data represents mean ± standard error of the mean of n=3 replicates.

[1] Cells were grown at 37 °C liquid culture with vigorous shaking, in minimal defined media with the indicated carbon source at 1% (w/v) concentration (see Materials and Methods: Metabolomic analysis by LC-MS).

[2] AMP signals were too low to be determined under these conditions.

Supplementary Table 3: Relative abundance of metabolites in wild type and pykΔectd cells grown in different carbon sources

strain		WT									*pyk Δectd*								
carbon source		glucose			malate			pyruvate			glucose			malate			pyruvate		
repeat		1	2	3	1	2	3	1	2	3	1	2	3	1	2	3	1	2	3
	normalization																		
Valine	normalized to 13C labeled internal control	4.242114	1.833274	1.969748	2.213543546	2.234399026	2.107623519	2.48392824	2.065160698	1.99758838	3.365482059	4.052190926	2.22630869	6.59599443	7.435774345	3.494954306	8.195901609	10.02652189	15.63859
UTP	normalized to 13C labeled internal control	22.73475	11.90729	8.367272	10.14787573	12.1841605	8.118915646	19.2062491	22.06261523	9.80124436	10.1335364	14.17232728	9.02726701	13.58132819	11.54640758	9.003617295	17.01173969	12.65382301	14.37581
Uridine	normalized to 13C labeled internal control	28.30768	6.705588	5.612767	6.214400369	7.872420608	4.731332196	4.205836133	3.067322485	4.91520201	37.4832147	4.771270133	5.77759451	5.094633972	5.071110602	19.91671644	6.361152527	9.051423864	7.575312
UDP-N-acetylglucosamine	normalized to 13C labeled internal control	2.110042	2.85587	3.017458	4.086290388	5.92004293	8.453462947	0.735004312	0.540799899	0.7025079	4.459472337	3.446487632	2.94585266	2.652048407	0	0	0.794842229	0.669443355	0.565637
UDP-glucuronate	normalized to 13C labeled internal control	3.228789	2.390232	1.594979	1.451663663	1.982461816	1.579764004	1.628684827	0.858113332	1.6678253	2.424364299	3.347160321	3.24613399	1.423922361	1.151193468	2.899299356	1.35578685	1.176603447	0.975074
Tyrosine	normalized to 13C labeled internal control	2.154293	2.380023	2.246076	2.031788303	2.557865723	1.720236338	1.522009063	1.733534669	1.67438527	3.086230754	1.772464626	1.58449771	2.006028347	1.656874229	3.125311654	2.266679348	3.309820753	3.62828
Threonine	normalized to 13C labeled internal control	1.067678	1.345354	1.294867	1.694549381	1.958419801	1.416851272	1.178639397	1.146588142	1.21573302	1.57438575	0.927669151	1.0902068	2.059327735	2.082741109	1.756908334	0.97769398	1.112500968	1.247854
Shikimate 3-phosphate	normalized to 13C labeled internal control	3.737943	6.174121	4.765636	0.72169147	0.595251357	0.494137397	0.242266919	0.335187399	0.07047827	3.117971154	4.42914118	6.47186777	0.813975915	0.424356744	1.112551244	0.088670712	0.031001749	0.369614
ribose-5-phosphate	normalized to 13C labeled internal control	2.413446	4.202278	3.368468	1.543952876	1.440958571	1.378586603	0.796300646	0.878691573	0.71166771	3.262727493	2.407235771	2.18274476	0.601691293	0.676592664	0.43274058	0.415911843	0.349454686	0.377449
ribulose-5-phosphate	normalized to 13C labeled internal control	2.316445	4.568393	4.740061	1.422841651	1.284169831	1.183919398	1.26265045	0.938745133	1.40600996	3.950461961	2.790882009	1.98929877	0.400015253	0.534536587	0.345269677	0.399548872	0.452505837	0.484458
pyruvate	normalized to 13C labeled internal control	12.37788	6.067836	45.13923	62.0062143	62.49344789	73.487851	1253.560253	2595.630226	2706.77067	7.471135797	6.937280135	32.5732262	93.63934131	149.6118782	58.11830052	1594.90515	1349.657804	1247.106
phosphoenolpyruvate	normalized to 13C labeled internal control	0.798025	2.234765	2.076791	126.7937907	139.7066096	72.49083204	53.86385813	48.81367284	57.7721336	3.559714673	1.840263712	0.79113759	6.099578924	17.07436395	9.755674607	15.2775501	8.132942124	9.455958
Leucine	normalized to 13C labeled internal control	4.185523	2.411782	2.603189	2.562307711	2.739276761	2.419283949	3.454476563	3.356309319	3.26440429	4.662459052	4.107085368	2.77523072	3.433387845	3.304021906	3.119487557	6.057846853	7.902888012	10.27029
Lactate	normalized to 13C labeled internal control	0.128171	0.17405	0.108487	0.614389889	0.570118667	0.698465266	0.104950455	0.080073049	0.09801446	0.141491889	0.133335755	0.16522154	0.57727801	0.654894072	0.271327758	0.110523235	0.148936193	0.195907
Isoleucine	normalized to 13C labeled internal control	2.858137	2.256204	2.072679	2.376046492	2.574634553	2.461794271	2.239758647	1.964542459	2.03308284	3.486402351	2.682461794	2.12998684	4.753174431	4.247529555	3.552874852	2.810416491	3.665667869	5.136979
Glyceraldeyde-3-Phosphate	normalized to OD600	16050.59	48292.49	39544.2	35343653.33	18260142.55	286840.5581	140114409.1	134572733.3	96570088	12144330.19	11234531.71	8464056.82	52518390.24	37160292.31	21899336.59	111916151.7	116853727.7	1.3E+08
Glutamate	normalized to 13C labeled internal control	1.788598	1.496517	1.492226	2.477002501	2.649825927	2.189955208	3.512523439	3.459001192	3.50728104	1.44871212	1.635485797	1.50170074	2.635962047	2.27888691	3.100866138	3.691448095	3.414434733	3.11451
Dihydroorotate	normalized to 13C labeled internal control	2.936883	1.711681	1.791769	1.532559402	1.534182413	1.665874942	0.679720002	0.603704329	0.57075281	1.671758488	1.828578917	2.87001498	0.94491503	1.164873649	0.477564802	0.721338876	0.930967882	0.644019
Citrulline	normalized to 13C labeled internal control	2.404406	0.745695	0.916796	0.790022047	1.614422127	1.334391117	1.113689281	1.051032043	1.0725863	0.907772303	2.207229553	2.12059161	1.22126195	1.09271976	1.853307694	1.161212395	1.127771244	1.114381
Citrate	normalized to 13C labeled internal control	2.885489	3.989396	3.365913	7.86795786	8.501881483	6.314140888	7.010038646	6.939672196	7.68703453	4.742428728	2.988128279	2.20174994	5.653398373	4.749185826	8.296571537	4.957346276	6.062483101	6.274397
Bisphospho-glycerate	normalized to OD600	26989.23	0	21754.71	12795457.78	12948267.02	11624220.93	6809529.545	5988050	3255132.5	398058.8491	53994.56098	0	497785.3659	2653260.256	548245.2439	1872253.879	543678.2553	1525606
ATP	normalized to 13C labeled internal control	9.927399	9.16695	7.159139	20.24203137	12.06158688	9.955696529	8.630300313	6.625909549	7.05218377	8.004450457	10.92032758	6.56079468	19.50807462	10.29520314	8.593953223	18.21995797	15.03698062	15.95097
Aspartic acid	normalized to 13C labeled internal control	0.571265	1.549895	1.583744	7.199762677	11.24760733	5.925540884	1.683226086	1.75291432	1.85839274	1.806949475	0.598297772	0.7211326	4.828325971	4.560519635	5.043993329	1.205939158	1.273928868	1.084404
alpha-ketoglutarate	normalized to 13C labeled internal control	0.075615	0.073734	0.075367	0.525343713	0.51176378	0.570023939	0.074231884	0.062609306	0.05114264	0.093294775	0.097253699	0.06351926	0.273096957	0.334144133	0.843176765	0.302085811	0.023259301	0.097192
Alanine	normalized to OD600	1384415	1032073	2803354	98485048.89	90928595.74	67637116.28	6891962.5	4012525.521	6722793	3833083.491	2092933.537	740572.659	144013824.4	155455179.5	115093365.9	65765289.66	84911404.26	1.69E+08
Aconitate	normalized to 13C labeled internal control	0.7501	0.688396	0.641813	1.660578865	1.743740373	1.402939747	1.234292963	1.318466045	1.47634898	0.901956009	0.84968573	0.58664547	0.988774718	1.073589808	2.230031945	0.08448645	0.920877939	0.88013
Acetyl-Ornithine	normalized to 13C labeled internal control	1.355136	0.675439	0.64508	9.557254241	8.260627231	5.566053899	4.248639208	7.652586414	9.90539544	0.652350593	2.315917887	1.75145756	12.90164264	4.356189106	12.37192256	9.486534602	13.03153576	9.433111
Acetyl Coenzyme A	normalized to 13C labeled internal control	3.411953	5.547999	3.785931	3.350503916	3.754297525	3.218157818	9.118057052	2.854457716	4.98596908	5.5440257	6.440776115	1.75086667	5.90735627	2.348007919	2.388993539	12.07109787	11.63586919	10.75167
5-Dehydroquinate	normalized to 13C labeled internal control	1.557661	2.913687	2.608884	2.074580776	2.154717189	1.988047204	1.899148214	1.828438541	2.21336825	3.070534213	1.759190131	1.47888309	1.608039276	1.905325921	1.564582954	1.886594146	1.794842243	1.611243
3-Phospho-Glycerate	normalized to OD600	2830216	13362153	23213221	168043733.3	367992510.6	195359497.7	58840527.27	36954945.83	74837080	58806279.25	44392468.29	21821100	11496531.71	21247712.82	23882968.29	30734355.17	7914231.915	7149149
Serine	normalized to 13C labeled internal control	71.79349	52.01975	40.86318	69.33525858	7.055639901	63.822116	70.10420983	5.847500813	66.6667333	66.73282075	65.54882799	72.5447374	71.23126021	76.02272554	72.51342866	44.28058639	2.777529414	60.23667
Sedheptulose 7-phosphate	normalized to OD600	30844364	73196953	60625621	10917822.22	12897391.49	510088.6512	3021011.932	2054686.333	2384250.76	61686184.91	35191190.24	36604068.2	2432330.78	29699.84615	1483358.244	68689.36207	505697.9362	84087.81
Riboflavin	normalized to OD600	13868368	9801530	10604626	95575.51111	114042.6596	159720.4884	179647.7727	149308.9167	124038.58	11354077.36	15732968.29	13051930.7	253586.3902	351482.2051	0	120018.931	240908.3404	269087.4
phenylpyruvate	normalized to 13C labeled internal control	3.366416	3.296352	3.032691	2.137505162	2.007526729	2.521610327	1.698195175	1.737410182	1.93628958	3.358315263	3.323808469	1.62225835	1.339857199	1.801867773	2.746985561	3.644225079	5.259209799	7.799969
Phenylalanine	normalized to 13C labeled internal control	3.613588	1.428579	3.328946	1.798049062	0.013545518	1.444313757	2.649317582	1.974759016	1.52124873	3.168067436	4.276500242	1.45411416	1.701640131	2.424992117	3.178948566	1.608995794	4.907474485	2.247786
Ornithine	normalized to 13C labeled internal control	2.255565	1.028725	0.939016	1.401810331	1.541138723	1.194586994	1.040915465	0.943129308	0.96539247	0.855725818	3.8983501	1.60933164	1.125388888	1.032545504	1.737288958	1.209845346	1.013951429	1.058992
NADPH	normalized to OD600	421184.8	699469.6	844643	3322067.778	1092869.277	2233448.977	2436941.182	109026.0417	1631938.24	744792.2264	1408900.927	820780.114	1511054.732	987675.5641	1515621.341	336260.3448	503523.8723	3148180
NADP+	normalized to OD600	160052.4	1487264	854574.1	12005196.67	20777212.77	0	2956352	1360530.458	2924699	6852512.736	6655901.829	6751888.64	19581854.88	5934512.821	14420978.05	10491113.79	11798251.06	6205330
NAD+	normalized to 13C labeled internal control	1.264613	1.831875	1.515706	1.821730475	2.230111698	1.904133014	2.425473083	1.37172209	1.70102044	1.80124114	1.358210101	1.66396239	1.91357457	1.497604821	1.568192359	2.297194704	1.746410846	1.717223
Myo-inositol	normalized to 13C labeled internal control	1.369964	1.35757	1.000344	0.004228575	0.002604058	0.004360043	0.010474843	0.004101768	0.00420611	1.345066069	1.519548364	1.17832614	0.012740415	0.004907139	0.004283388	0.003130954	0.004122335	0.00387
Methionine	normalized to 13C labeled internal control	1.136552	1.054989	2.637959	2.512013191	2.025719633	2.484685902	1.377184922	1.235383232	1.59402794	6.765546737	1.967145169	1.20070776	6.101285501	1.671177513	2.2017583	0.869024875	1.88273251	2.219062
MEP	normalized to OD600	29816905	30229528	19566696	41481182.22	35030212.77	40422837.21	49541263.64	31046864.58	24036664	39425611.32	32360790.24	40513868.2	39724348.78	47934338.46	44850746.34	27614563.79	29185770.21	29250353
Malonyl-CoA	normalized to OD600	344947.1	813094.8	835615.2	12802367.78	12877288.3	16423403.49	8802140.909	500835.0208	4893691.5	7570817.453	2709622.244	114330.477	16751262.2	8723154.487	19226974.39	32432800	19922917.02	10966297
Malate	normalized to OD600	662894.2	515253.2	352151.3	6.63E+11	6.41E+11	7.90E+11	1824376.136	691669.2708	1222346.38	0	854621.9512	14002836.4	6.50E+11	5.28E+11	3.93E+11	11218655.17	7561153.191	11479694
Histidine	normalized to OD600	2.92E+08	2.34E+08	2.09E+08	401938204.4	372973004.3	433698530.2	447267563.6	384545233.3	367294176	205222550.9	283443882.9	261213255	177889053.7	466011405.1	423395122	314812413.8	374999761.7	1.68E+08
GTP	normalized to OD600	135534.7	2781950	2799844	6062412.222	18560429.79	19199875.58	3891175.864	72923.625	0	6795534.434	11716187.8	4548909.66	13727529.27	3157867.308	3217600.927	32043213.79	8182359.043	27218985
Glycerol-3-phosphate	normalized to OD600	33420395	33912864	27575193	32078022.22	33135893.62	35441376.74	25138109.09	20101297.92	27078848	48907758.49	45944356.1	31069800	35626809.76	38415456.41	43933848.78	26251796.55	32752738.3	34720485
Glutamine	normalized to 13C labeled internal control	1.835058	1.202891	2.227149	3.452458235	3.589891503	2.830929373	2.530087255	1.503858101	1.35390191	0.687994783	1.446968804	1.11458595	2.474558439	4.788892139	14.2044191	3.908878028	3.914484748	2.894455
Glucose-6-phosphate	normalized to 13C labeled internal control	2.440874	5.807723	5.178889	0.74211565	0.734972553	0.649136281	0.478050745	0.408871599	0.43309323	5.039199806	2.538885859	2.28877253	0.304139218	0.389505459	0.526971679	0.324677853	0.258154236	0.379627
GGPP	normalized to OD600	3465255	3603450	3996271	4373393.6	5715707.979	5109924.419	3914028.977	4766854.688	3937246	3567790.566	5185533.537	3917966.77	7028767.683	5895616.667	4175860.366	3227268.759	5474223.936	4512470
Fumarate	normalized to OD600	67384133	44727719	44878204	11556196124	10704191183	13348304372	1226330618	1024895467	1285262080	42914181.13	60062034.15	54332277.3	11606695337	11306795323	11237317307	1113786262	1272096136	1.19E+09
Fructose 1_6-bisphosphate	normalized to OD600	2383499	69110477	93593786	453626.2222	763334.1064	1072990.837	186421.2045	75610.25	30205.18	69562649.06	113878848.8	62424795.5	3537531.415	515931.9231	0	302712.6379	102951.3404	2221563
FAD	normalized to OD600	4178353	1474050	1188839	1897324.444	3499917.553	3643725	1264692.045	0	0	1575813.925	3135114.024	2648334.66	4212617.366	635713.5385	1422819.366	2772151.517	1652003.319	1692522
dTTP	normalized to OD600	1438507	8700582	7414535	6019007.778	8161903.191	8019089.535	4797472.159	1537777.479	3459660.24	13662701.89	14616139.02	8659659.09	7344964.634	3065200	4751078.049	9175745.69	8163577.66	9917818
Dihydroxyacetone phosphate	normalized to 13C labeled internal control	2.363088	3.707597	3.937908	2.521443408	2.596415611	2.125224864	1.837191251	1.705298254	1.86064154	4.306267809	3.299096347	1.60887174	1.672764352	2.1174719	3.203144741	2.232711007	1.998262927	1.9429
Deoxyribose-5-phosphate	normalized to 13C labeled internal control	4.107429	4.712014	5.017141	2.810051756	4.02108214	3.513941928	2.672625459	4.389451264	4.53786479	3.23679701	1.651084078	1.92108231	3.454713205	2.338543833	2.502683508	4.290646436	3.469407164	2.393929
dCTP	normalized to OD600	1064735	4131546	3311061	2652663.333	3047775.532	3215199.116	1126228.045	62111.625	80304.76	5418499.528	8868813.415	3145277.84	5971237.805	4091321.154	108673.439	3136058.621	1201268.34	1480187
CTP	normalized to OD600	149264.1	3120589	5110090	4577221.667	18428304.26	14065205.81	4017676.136	637538.0833	2661253	4426111.321	10070976.22	3629214.77	15276821.95	4852259.949	5648457.317	11675236.21	4561546.809	8359216
cMEPP	normalized to 13C labeled internal control	4.157839	8.101886	7.974264	4.863542091	4.878335999	4.08708447	5.265237948	4.14899383	4.84322806	8.076620745	4.085733569	2.98244663	3.061433513	2.564026862	16.00380989	4.40090802	3.716762096	2.490925
CDP-MEP	normalized to OD600	229378.2	1262180	1356662	1675802.356	1757996.553	408842.9767	62897.72727	63236.3125	67961.78	1800930.075	405732.6341	361421.636	451865.9268	55342.64103	1588604.268	53034.01724	286557.0426	49715.85
Asparagine	normalized to OD600	63208564	46905864	46887921	81414933.33	78691982.98	89581618.6	96318681.82	77932316.67	78795168	46152381.13	58068004.88	58561868.2	95461112.2	101953753.8	84532663.41	65389951.72	82400017.02	76583140
AMP	normalized to OD600	4792715	3787912	2010923	0	0	0	6962669.318	3282500.521	6106852.5	1498963.434	5403845.732	15815265.9	647079.0488	0	312742.7073	5361390.517	3795619.404	2595723
Acetyl phosphate	normalized to OD600	4358054	10899494	14821291	10599401.11	7764777.66	8148201.163	7690973.295	5617385.417	4249854	19042001.89	10543926.83	5302788.64	8146360.976	11304873.08	7812787.805	5215312.069	2649794.681	4167988
3-Dehydroshikimate	normalized to OD600	2.559393	6.22578	6.480556	12.34337708	11.04875121	12.2131306	5.545877533	5.59191292	7.27909779	8.926876265	2.670706783	1.75804021	6.884202603	7.960931311	7.37703602	6.949754602	6.183996888	6.273554
3-Dehydroquinate	normalized to OD600	2390378	4113259	7777988	12967415.56	14551884.04	11430705.81	5221782.955	6628032.292	6678764.5	15836047.17	3125884.439	1199060.23	9675697.561	12462807.69	160081.8293	4308818.103	4296904.511	3371528
2_3-Dihydrobenzoic acid	normalized to OD600	3.2E+08	1.44E+09	1.06E+09	115475164.4	127423046.8	81627572.09	8662944.318	3135383.333	17262298	2926543940	691837736.6	110347873	39998239.02	0	119041014.6	15532951.72	11466423.4	8300145
